# Supplementary material for: Drosophila SUMM4 complex couples insulator function and DNA replication control
Source: eLife. 2022 Dec 2;11:e81828. doi: 10.7554/eLife.81828 (PMC9917439; doi:10.7554/eLife.81828)
Supplement: Figure 1—source data 1. — The following FPLC column parameters were used for partial purification of native SUMM4. HEG: 25 mM HEPES, pH 7.6, 0.1 mM EDTA, 10% glycerol, 0.02% NP-40, 1 mM DTT, 1 mM benzamidine, 0.4 mM PMSF; 10 mM KPi: 10 mM potassium phosphate, pH 7.6, 10% glycerol, 1 mM DTT, 1 mM benzamidine, 0.4 mM PMSF; 0.8 M KPi: 800 mM potassium phosphate, pH 7.6, 10% glycerol, 1 mM DTT, 1 mM benzamidine, 0.4 mM PMSF; cv, column volume. [file elife-81828-fig1-data1.docx]

***Figure 1⎯source data 1.*** FPLC column parameters (***Figure 1A***). The following FPLC column parameters were used for partial purification of native SUMM4. HEG: 25 mM HEPES, pH 7.6, 0.1 mM EDTA, 10% glycerol, 0.02% NP-40, 1 mM DTT, 1 mM benzamidine, 0.4 mM PMSF; 10 mM KPi: 10 mM potassium phosphate, pH 7.6, 10% glycerol, 1 mM DTT, 1 mM benzamidine, 0.4 mM PMSF; 0.8 M KPi: 800 mM potassium phosphate, pH 7.6, 10% glycerol, 1 mM DTT, 1 mM benzamidine, 0.4 mM PMSF; *cv*, column volume.

| **Column** | **Phosphocellulose** | **Source 15Q** | **Source 15S** | **Superdex 200 Increase** | **Hydroxylapatite** |
| --- | --- | --- | --- | --- | --- |
| Column volume, ml | 48 | 4 | 0.5 | 24 | 0.5 |
| Buffer A | HEG | HEG | HEG | HEG + 0.15 M NaCl | 10 mM KPi |
| Buffer B | HEG + 1 M NaCl | HEG + 1 M NaCl | HEG + 1 M NaCl | N/A | 0.8 M KPi |
| Starting material (SM) | nuclear extract | fxns 3-7 (Ph-Cell) | fxn 7 (15Q) | fxns 8-12 (15S) | fxns 10-11 (Superdex) |
| SM volume, ml | 100 | 38 | 1.2 | 0.6 | 0.9 |
| Diluted with | Buffer A | Buffer A | Buffer A | N/A | Buffer A |
| Dilution volume, ml | 50 | 85 | 5 | N/A | 1.8 |
| Equilibrate to, %B | 10% | 5% | 5% | 0% | 0% |
| Column wash, cv | 3 | 10 | 10 | N/A | 10 |
| Elution gradient | 10-100% | 5-100% | 5-100% | N/A | 0-100% |
| Elution volume, cv | 8 | 10 | 16 | 1.2 | 10 |
| Fraction volume, ml | 12 | 1.4 | 0.15 | 0.5 | 0.15 |
