# Supplementary figures and images for: Drosophila SUMM4 complex couples insulator function and DNA replication control
Source: eLife. 2022 Dec 2;11:e81828. doi: 10.7554/eLife.81828 (PMC9917439; doi:10.7554/eLife.81828)

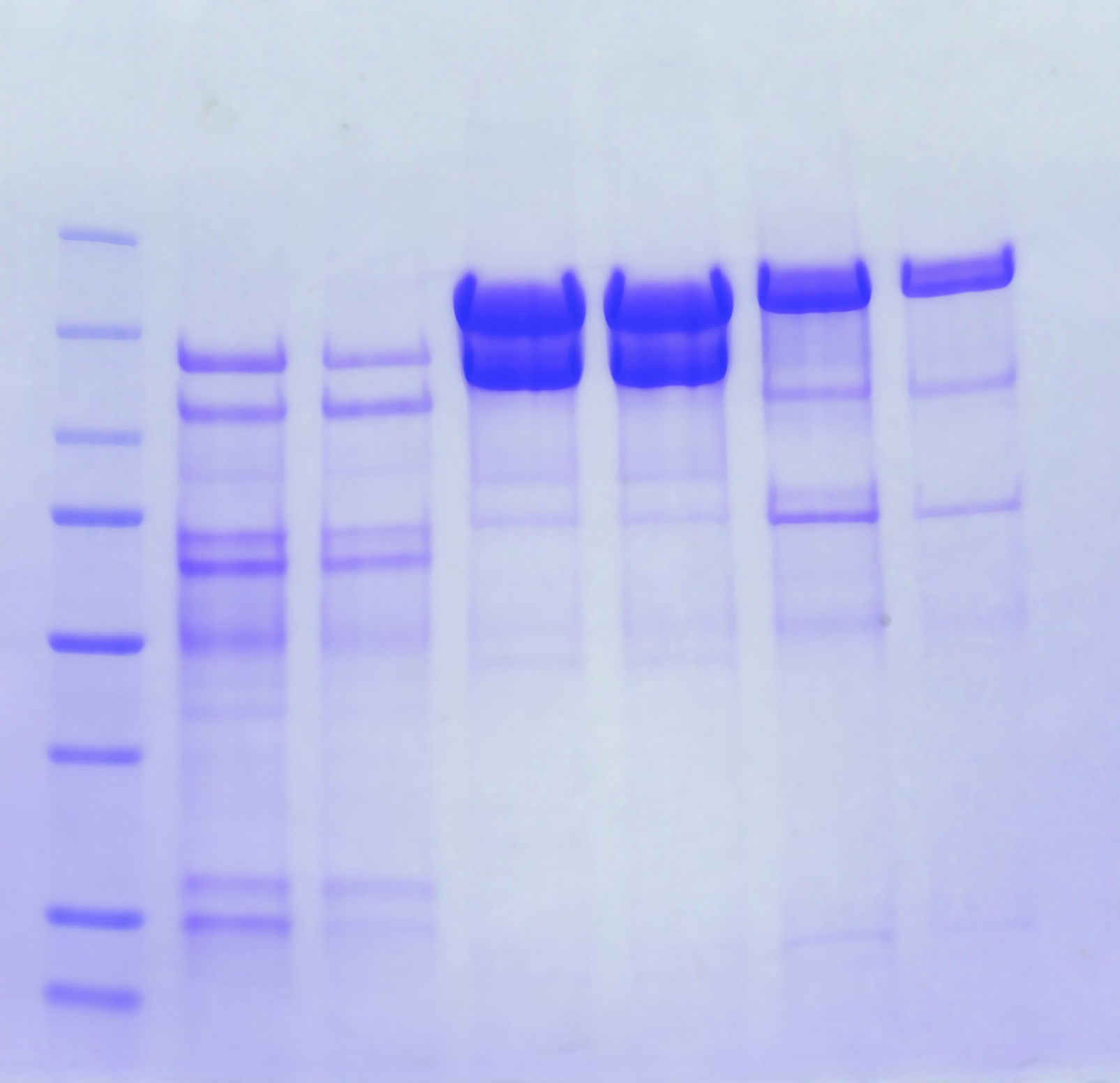

Supplement: Figure 1—source data 2. — Lane 1, protein size marker; lane 2, FLAG-SUUR, 72 hr infection of Sf9 cells; lane 3, FLAG-SUUR, 60 hr infection of Sf9 cells; lane 4, XNP-FLAG (Emelyanov et al., 2010), 72 hr infection of Sf9 cells; lane 5, XNP-FLAG, 60 hr infection of Sf9 cells; lane 6, EGG-FLAG, 72 hr infection of Sf9 cells; lane 7, EGG-FLAG, 60 hr infection of Sf9 cells. Prep amounts equivalent to ~20 ml Sf9 culture were loaded in each lane. Cropped images encompassing lanes 1–2 and 6 (open boxes, dashed red line) were used for Figure 1C. [file elife-81828-fig1-data2.zip › Figure 1-source data 2/8_14_20.jpg]

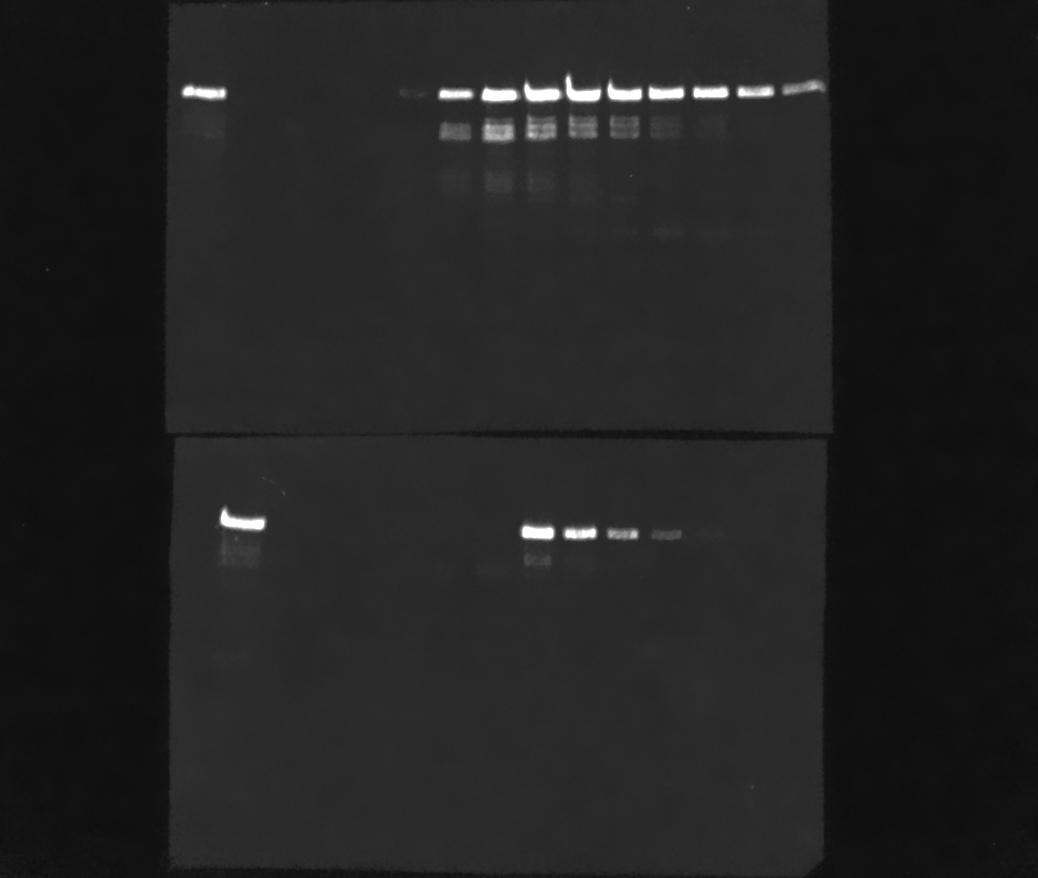

Supplement: Figure 2—source data 1. — Left panels, 700 nm channel (Odyssey Fc), rabbit anti-SUUR antibody and protein size marker; right panels, 800 nm channel (Odyssey Fc), guinea pig ModT antibody; top panels, hydroxylapatite fractions: starting material, flow-through, marker, fractions 1–12 (Figure 1H); bottom panels, Superdex 200 increase fractions: starting material, marker, fractions 5–15 (Figure 1G). Cropped images from bottom panels (open boxes, dashed red line) were used for Figure 2D. [file elife-81828-fig2-data1.zip › Figure 2-source data 1/0012748_01_800.jpg]

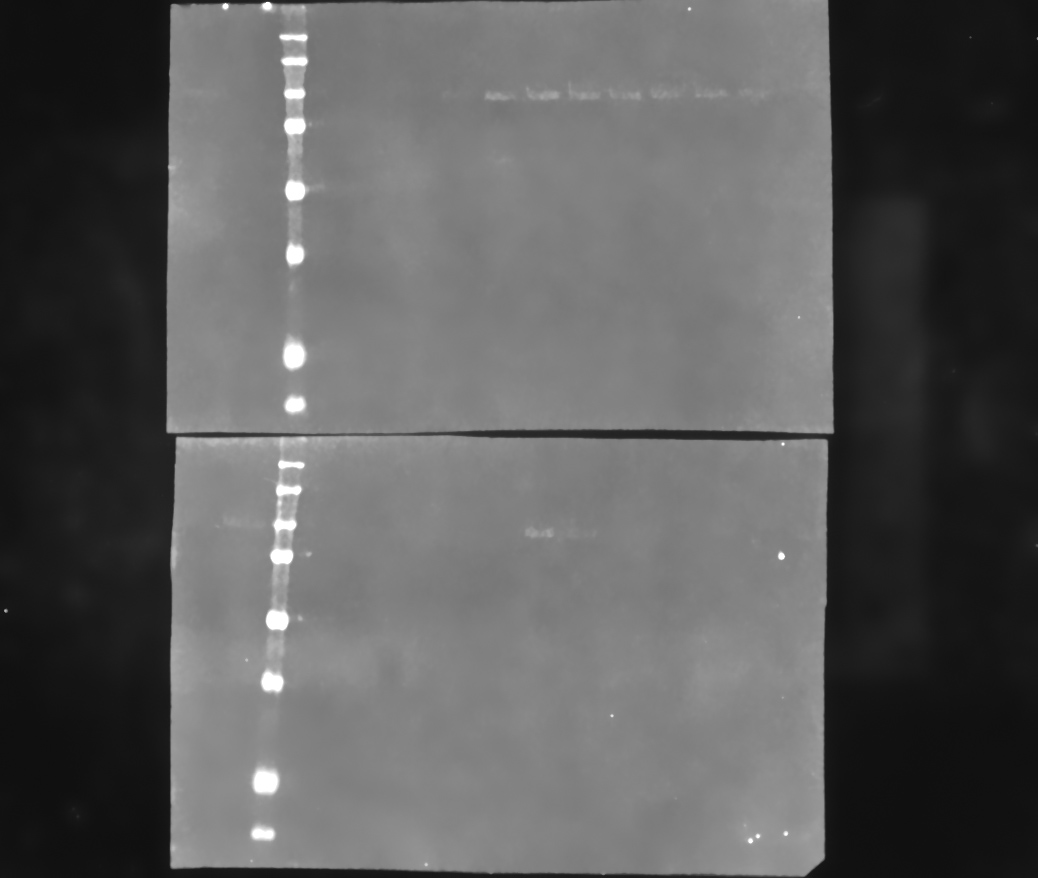

Supplement: Figure 2—source data 1. — Left panels, 700 nm channel (Odyssey Fc), rabbit anti-SUUR antibody and protein size marker; right panels, 800 nm channel (Odyssey Fc), guinea pig ModT antibody; top panels, hydroxylapatite fractions: starting material, flow-through, marker, fractions 1–12 (Figure 1H); bottom panels, Superdex 200 increase fractions: starting material, marker, fractions 5–15 (Figure 1G). Cropped images from bottom panels (open boxes, dashed red line) were used for Figure 2D. [file elife-81828-fig2-data1.zip › Figure 2-source data 1/0012748_01_700.jpg]

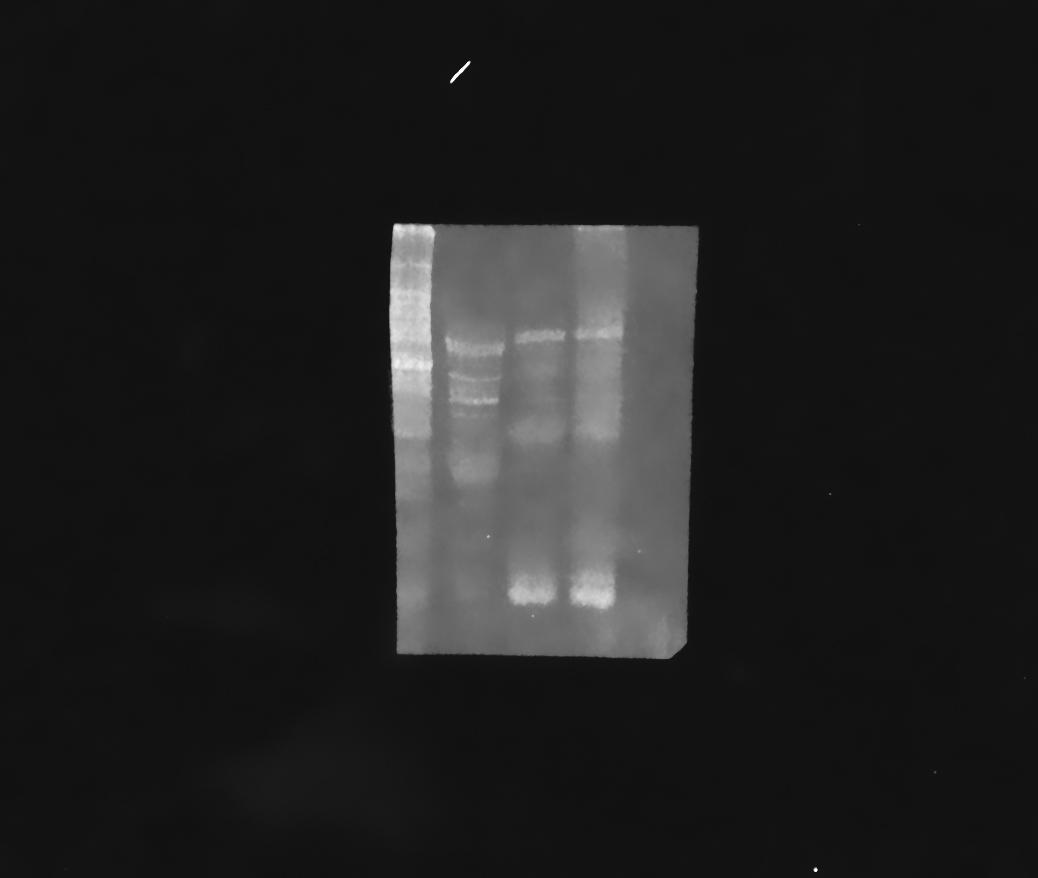

Supplement: Figure 2—source data 2. — (A, E) Westerns, 700 nm channel (Odyssey Fc), mouse anti-HP1a and protein size marker; (B) western, 800 nm channel (Odyssey Fc), rabbit anti-Mod(Mdg4)-FL; (C, G) Westerns, 700 nm channel (Odyssey Fc), protein sizemarker only; (D) Western, 800 nm channel (Odyssey Fc), rabbit anti-SUUR; (F) Western, 800 nm channel (Odyssey Fc), guinea pig ModT; (H) Western, 800 nm channel (Odyssey Fc), guinea pig anti-SUUR. Lanes 1, 5, 9, 12, 15, and 18, protein size marker; lanes 2, 6, 10, 13, 16, and 19, input (nuclear extract), 5 or 10%; lanes 3 and 7, IP with guinea pig ModT antibody #1; lanes 4 and 8, IP with guinea pig ModT antibody #2; lanes 11 and 17, IP with rabbit preimmune serum; lanes 14 and 20, IP with rabbit anti-XNP. Cropped images encompassing lanes 1–3, 5–7, 12–14, and 18–20 (open boxes, dashed red line) were used for Figure 2E. [file elife-81828-fig2-data2.zip › Figure 2-source data 2/0012718_01_800.jpg]

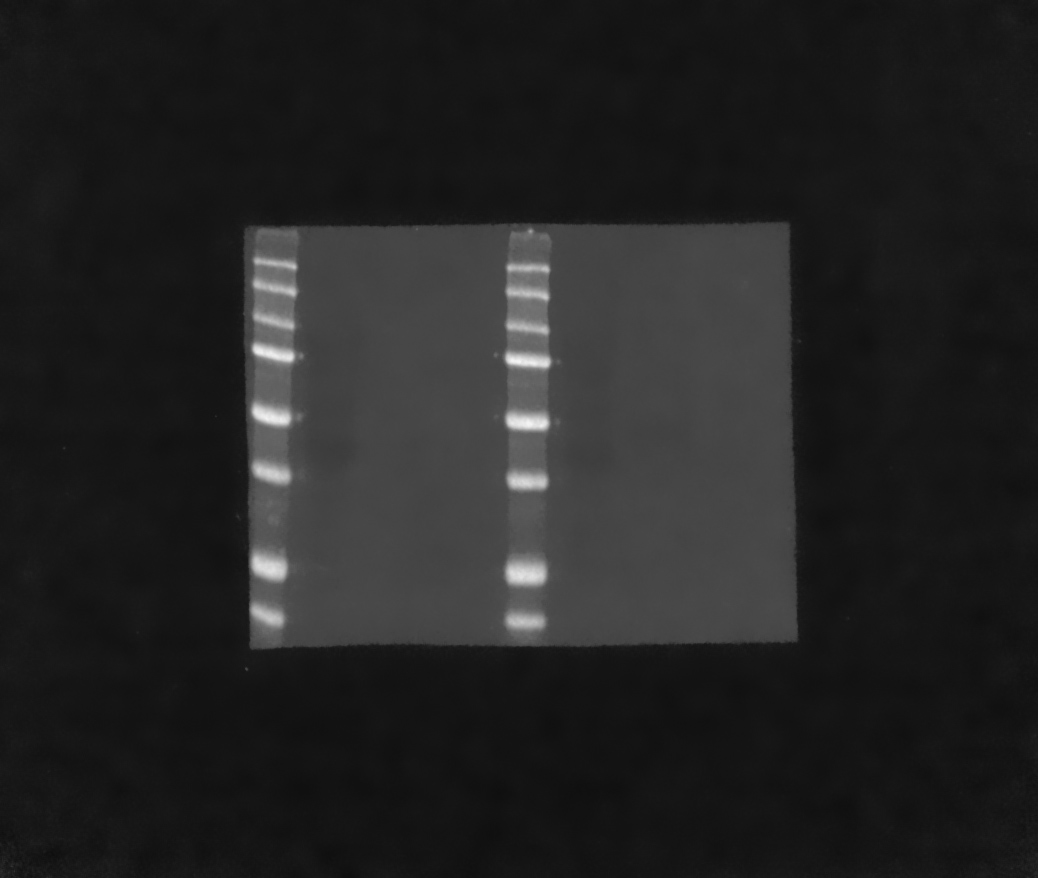

Supplement: Figure 2—source data 2. — (A, E) Westerns, 700 nm channel (Odyssey Fc), mouse anti-HP1a and protein size marker; (B) western, 800 nm channel (Odyssey Fc), rabbit anti-Mod(Mdg4)-FL; (C, G) Westerns, 700 nm channel (Odyssey Fc), protein sizemarker only; (D) Western, 800 nm channel (Odyssey Fc), rabbit anti-SUUR; (F) Western, 800 nm channel (Odyssey Fc), guinea pig ModT; (H) Western, 800 nm channel (Odyssey Fc), guinea pig anti-SUUR. Lanes 1, 5, 9, 12, 15, and 18, protein size marker; lanes 2, 6, 10, 13, 16, and 19, input (nuclear extract), 5 or 10%; lanes 3 and 7, IP with guinea pig ModT antibody #1; lanes 4 and 8, IP with guinea pig ModT antibody #2; lanes 11 and 17, IP with rabbit preimmune serum; lanes 14 and 20, IP with rabbit anti-XNP. Cropped images encompassing lanes 1–3, 5–7, 12–14, and 18–20 (open boxes, dashed red line) were used for Figure 2E. [file elife-81828-fig2-data2.zip › Figure 2-source data 2/0012730_01_700.jpg]

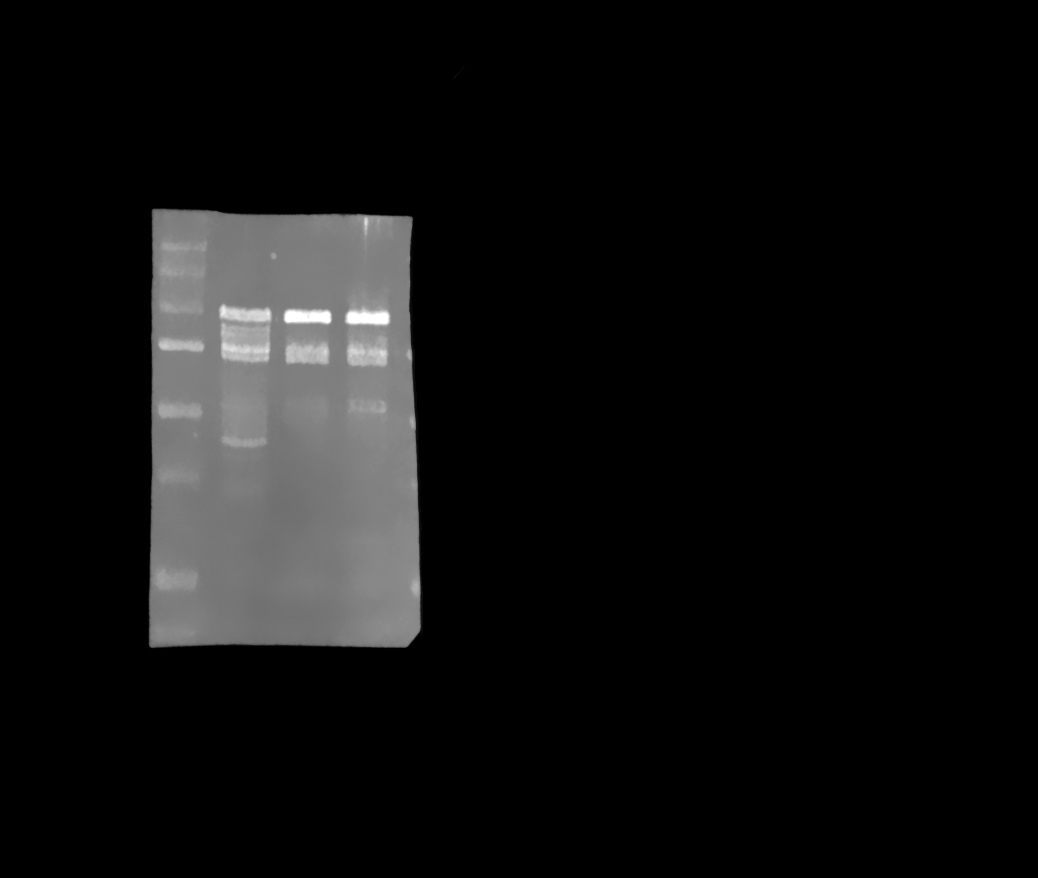

Supplement: Figure 2—source data 2. — (A, E) Westerns, 700 nm channel (Odyssey Fc), mouse anti-HP1a and protein size marker; (B) western, 800 nm channel (Odyssey Fc), rabbit anti-Mod(Mdg4)-FL; (C, G) Westerns, 700 nm channel (Odyssey Fc), protein sizemarker only; (D) Western, 800 nm channel (Odyssey Fc), rabbit anti-SUUR; (F) Western, 800 nm channel (Odyssey Fc), guinea pig ModT; (H) Western, 800 nm channel (Odyssey Fc), guinea pig anti-SUUR. Lanes 1, 5, 9, 12, 15, and 18, protein size marker; lanes 2, 6, 10, 13, 16, and 19, input (nuclear extract), 5 or 10%; lanes 3 and 7, IP with guinea pig ModT antibody #1; lanes 4 and 8, IP with guinea pig ModT antibody #2; lanes 11 and 17, IP with rabbit preimmune serum; lanes 14 and 20, IP with rabbit anti-XNP. Cropped images encompassing lanes 1–3, 5–7, 12–14, and 18–20 (open boxes, dashed red line) were used for Figure 2E. [file elife-81828-fig2-data2.zip › Figure 2-source data 2/0012717_01_800.jpg]

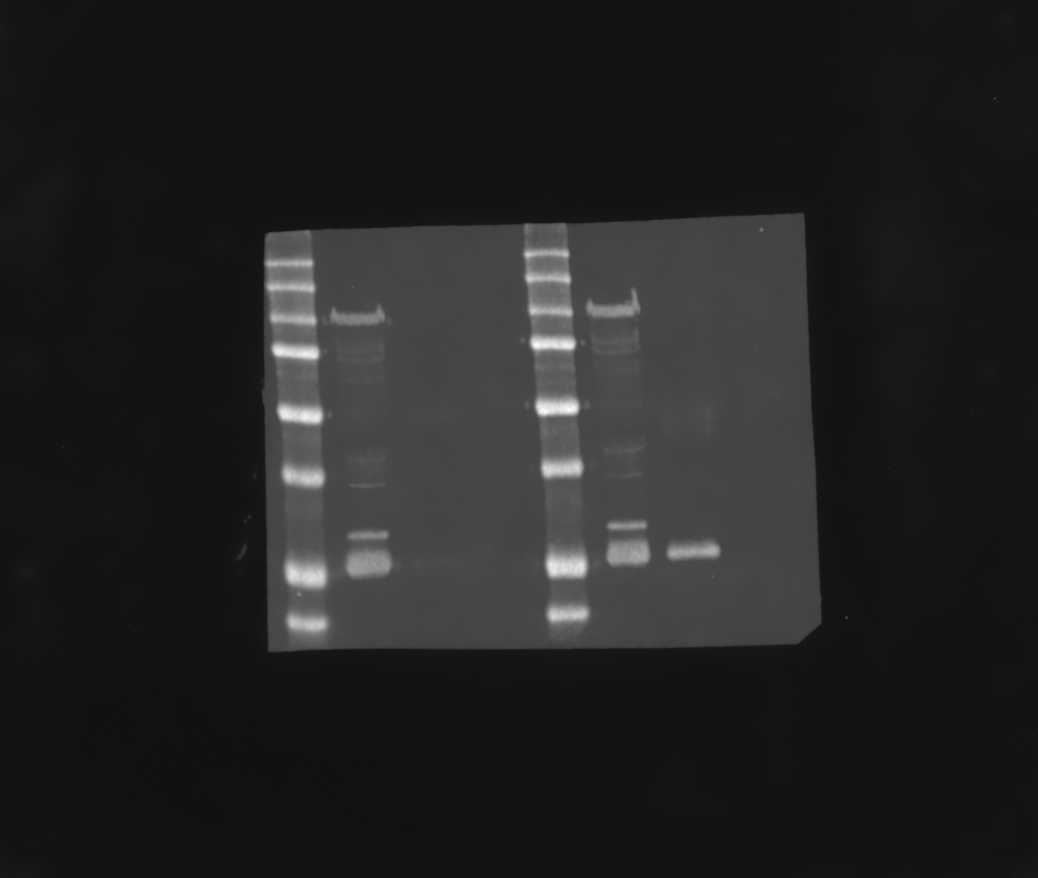

Supplement: Figure 2—source data 2. — (A, E) Westerns, 700 nm channel (Odyssey Fc), mouse anti-HP1a and protein size marker; (B) western, 800 nm channel (Odyssey Fc), rabbit anti-Mod(Mdg4)-FL; (C, G) Westerns, 700 nm channel (Odyssey Fc), protein sizemarker only; (D) Western, 800 nm channel (Odyssey Fc), rabbit anti-SUUR; (F) Western, 800 nm channel (Odyssey Fc), guinea pig ModT; (H) Western, 800 nm channel (Odyssey Fc), guinea pig anti-SUUR. Lanes 1, 5, 9, 12, 15, and 18, protein size marker; lanes 2, 6, 10, 13, 16, and 19, input (nuclear extract), 5 or 10%; lanes 3 and 7, IP with guinea pig ModT antibody #1; lanes 4 and 8, IP with guinea pig ModT antibody #2; lanes 11 and 17, IP with rabbit preimmune serum; lanes 14 and 20, IP with rabbit anti-XNP. Cropped images encompassing lanes 1–3, 5–7, 12–14, and 18–20 (open boxes, dashed red line) were used for Figure 2E. [file elife-81828-fig2-data2.zip › Figure 2-source data 2/0012729_01_700.jpg]

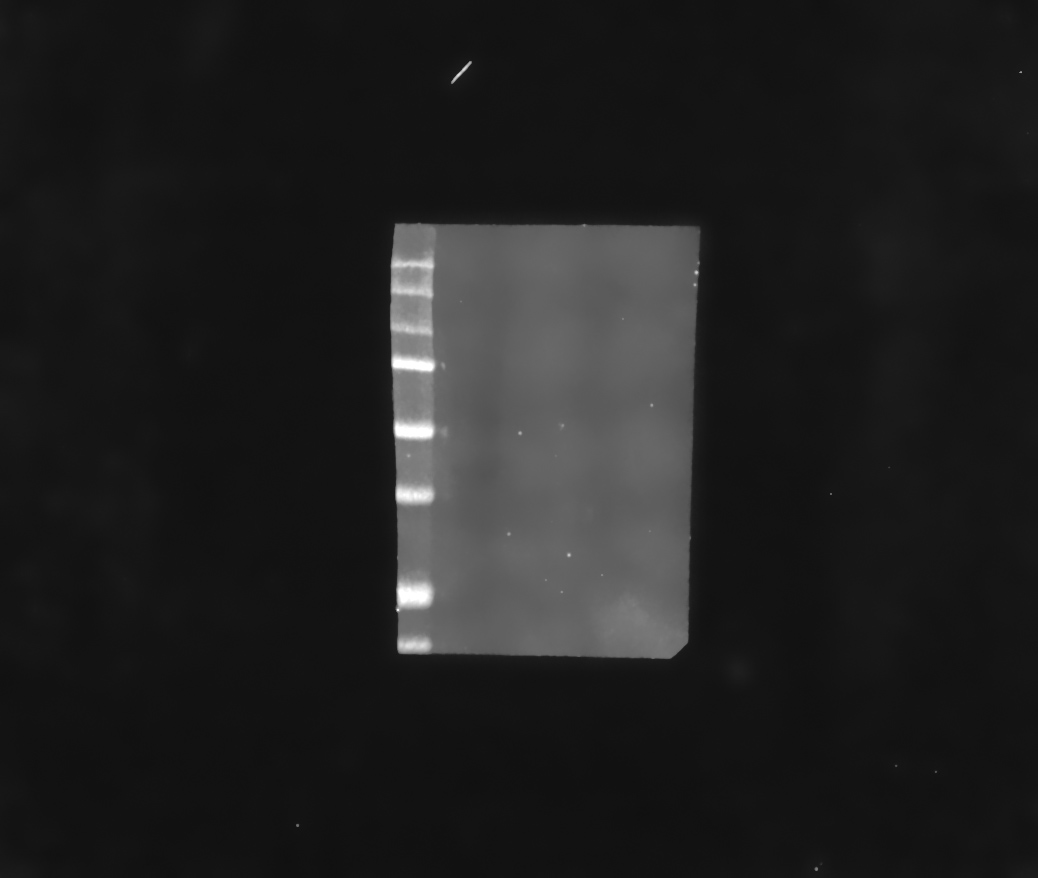

Supplement: Figure 2—source data 2. — (A, E) Westerns, 700 nm channel (Odyssey Fc), mouse anti-HP1a and protein size marker; (B) western, 800 nm channel (Odyssey Fc), rabbit anti-Mod(Mdg4)-FL; (C, G) Westerns, 700 nm channel (Odyssey Fc), protein sizemarker only; (D) Western, 800 nm channel (Odyssey Fc), rabbit anti-SUUR; (F) Western, 800 nm channel (Odyssey Fc), guinea pig ModT; (H) Western, 800 nm channel (Odyssey Fc), guinea pig anti-SUUR. Lanes 1, 5, 9, 12, 15, and 18, protein size marker; lanes 2, 6, 10, 13, 16, and 19, input (nuclear extract), 5 or 10%; lanes 3 and 7, IP with guinea pig ModT antibody #1; lanes 4 and 8, IP with guinea pig ModT antibody #2; lanes 11 and 17, IP with rabbit preimmune serum; lanes 14 and 20, IP with rabbit anti-XNP. Cropped images encompassing lanes 1–3, 5–7, 12–14, and 18–20 (open boxes, dashed red line) were used for Figure 2E. [file elife-81828-fig2-data2.zip › Figure 2-source data 2/0012718_01_700.jpg]

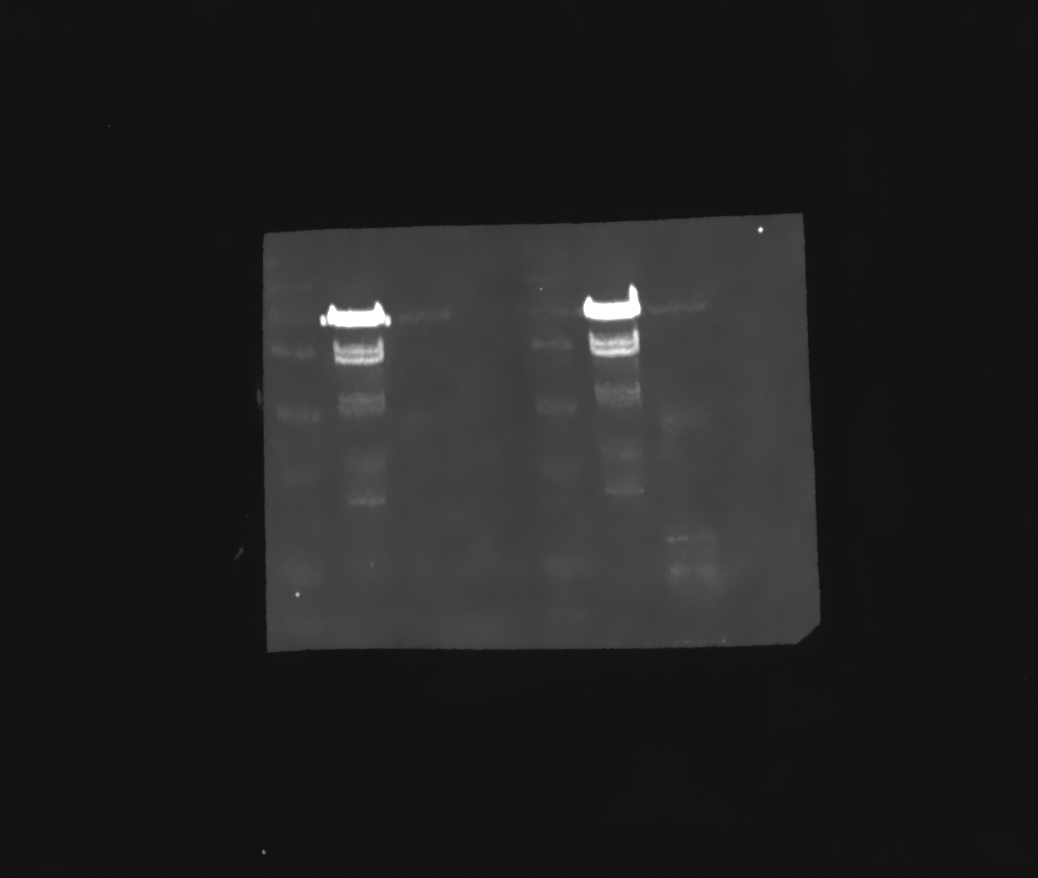

Supplement: Figure 2—source data 2. — (A, E) Westerns, 700 nm channel (Odyssey Fc), mouse anti-HP1a and protein size marker; (B) western, 800 nm channel (Odyssey Fc), rabbit anti-Mod(Mdg4)-FL; (C, G) Westerns, 700 nm channel (Odyssey Fc), protein sizemarker only; (D) Western, 800 nm channel (Odyssey Fc), rabbit anti-SUUR; (F) Western, 800 nm channel (Odyssey Fc), guinea pig ModT; (H) Western, 800 nm channel (Odyssey Fc), guinea pig anti-SUUR. Lanes 1, 5, 9, 12, 15, and 18, protein size marker; lanes 2, 6, 10, 13, 16, and 19, input (nuclear extract), 5 or 10%; lanes 3 and 7, IP with guinea pig ModT antibody #1; lanes 4 and 8, IP with guinea pig ModT antibody #2; lanes 11 and 17, IP with rabbit preimmune serum; lanes 14 and 20, IP with rabbit anti-XNP. Cropped images encompassing lanes 1–3, 5–7, 12–14, and 18–20 (open boxes, dashed red line) were used for Figure 2E. [file elife-81828-fig2-data2.zip › Figure 2-source data 2/0012729_01_800.jpg]

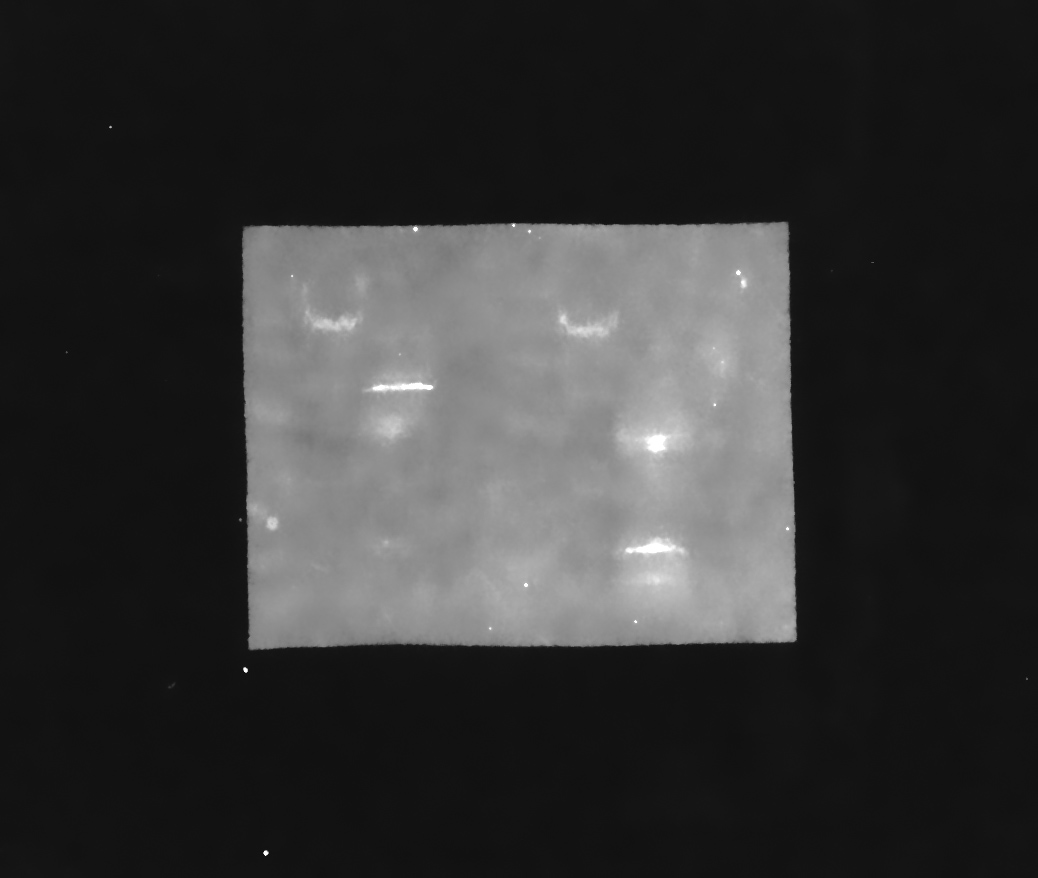

Supplement: Figure 2—source data 2. — (A, E) Westerns, 700 nm channel (Odyssey Fc), mouse anti-HP1a and protein size marker; (B) western, 800 nm channel (Odyssey Fc), rabbit anti-Mod(Mdg4)-FL; (C, G) Westerns, 700 nm channel (Odyssey Fc), protein sizemarker only; (D) Western, 800 nm channel (Odyssey Fc), rabbit anti-SUUR; (F) Western, 800 nm channel (Odyssey Fc), guinea pig ModT; (H) Western, 800 nm channel (Odyssey Fc), guinea pig anti-SUUR. Lanes 1, 5, 9, 12, 15, and 18, protein size marker; lanes 2, 6, 10, 13, 16, and 19, input (nuclear extract), 5 or 10%; lanes 3 and 7, IP with guinea pig ModT antibody #1; lanes 4 and 8, IP with guinea pig ModT antibody #2; lanes 11 and 17, IP with rabbit preimmune serum; lanes 14 and 20, IP with rabbit anti-XNP. Cropped images encompassing lanes 1–3, 5–7, 12–14, and 18–20 (open boxes, dashed red line) were used for Figure 2E. [file elife-81828-fig2-data2.zip › Figure 2-source data 2/0012730_01_800.jpg]

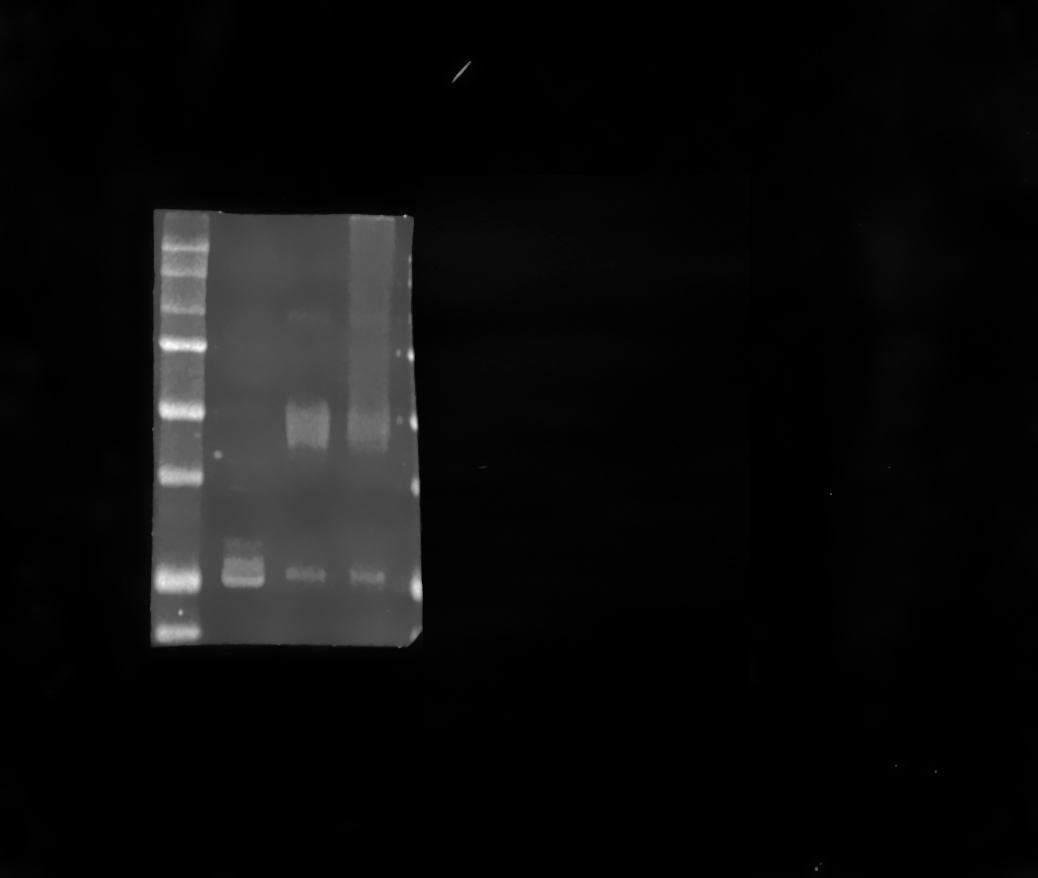

Supplement: Figure 2—source data 2. — (A, E) Westerns, 700 nm channel (Odyssey Fc), mouse anti-HP1a and protein size marker; (B) western, 800 nm channel (Odyssey Fc), rabbit anti-Mod(Mdg4)-FL; (C, G) Westerns, 700 nm channel (Odyssey Fc), protein sizemarker only; (D) Western, 800 nm channel (Odyssey Fc), rabbit anti-SUUR; (F) Western, 800 nm channel (Odyssey Fc), guinea pig ModT; (H) Western, 800 nm channel (Odyssey Fc), guinea pig anti-SUUR. Lanes 1, 5, 9, 12, 15, and 18, protein size marker; lanes 2, 6, 10, 13, 16, and 19, input (nuclear extract), 5 or 10%; lanes 3 and 7, IP with guinea pig ModT antibody #1; lanes 4 and 8, IP with guinea pig ModT antibody #2; lanes 11 and 17, IP with rabbit preimmune serum; lanes 14 and 20, IP with rabbit anti-XNP. Cropped images encompassing lanes 1–3, 5–7, 12–14, and 18–20 (open boxes, dashed red line) were used for Figure 2E. [file elife-81828-fig2-data2.zip › Figure 2-source data 2/0012717_01_700.jpg]

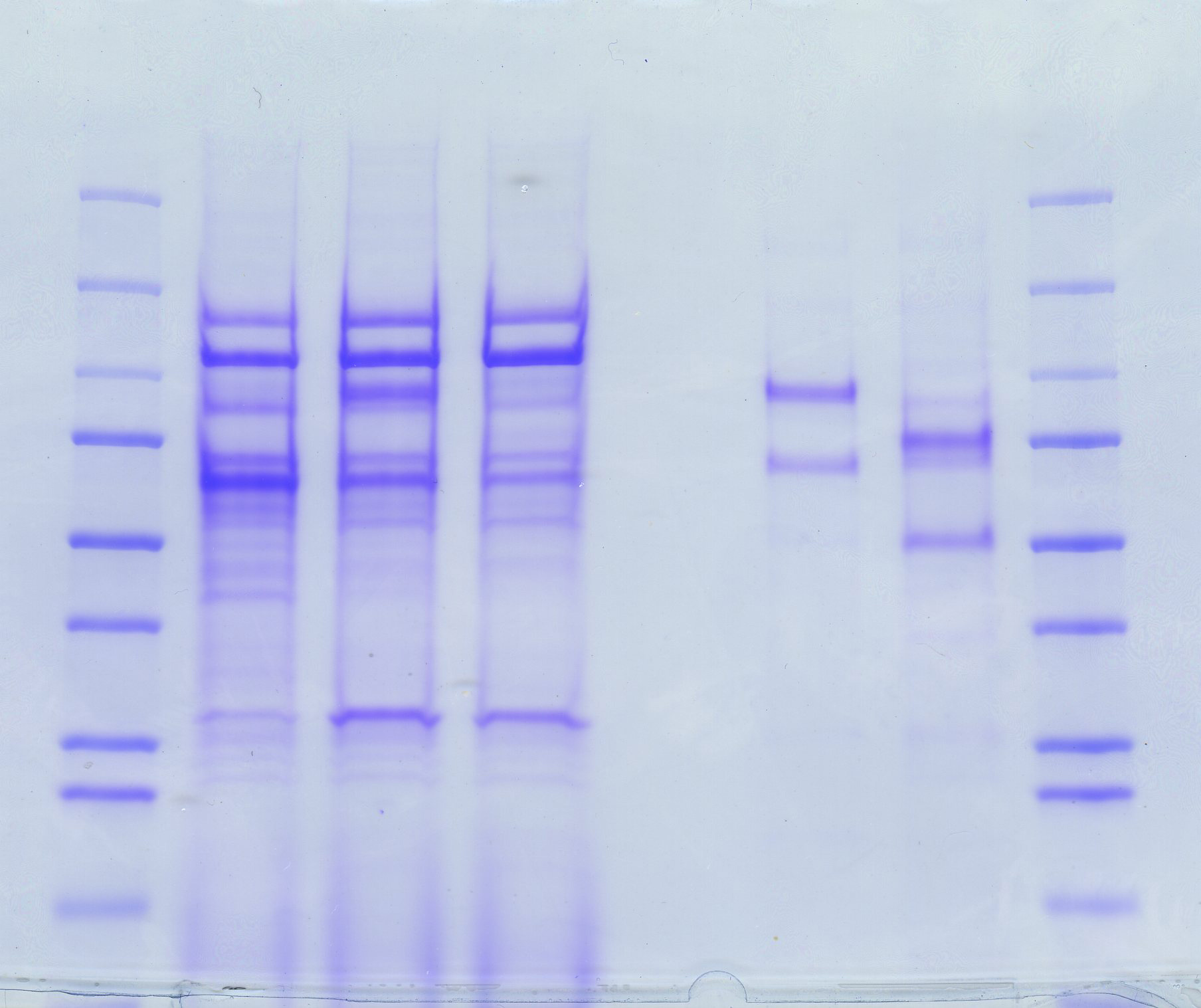

Supplement: Figure 3—source data 1. — Lanes 1 and 7, protein size marker; lane 2, FLAG-SUUR, FLAG-purified; lane 3, FLAG-SUUR + Mod(Mdg4)-67.2-His6, FLAG-purified; lane 4, FLAG-SUUR + Mod(Mdg4)-59.1-His6, FLAG-purified; lane 5, Mod(Mdg4)-67.2-His6, Ni-NTA-purified; lane 6, Mod(Mdg4)–-9.1-His6, Ni-NTA-purified. All proteins were purified 72 hr post-infection. Prep amounts equivalent to ~20 ml (FLAG-purified, lanes 2–4) or ~1 ml (Ni-NTA-purified, lanes 5 and 6) Sf9 cultures were loaded in each lane. Cropped image encompassing all lanes (open box, dashed red line) was used for Figure 3A. [file elife-81828-fig3-data1.zip › Figure 3-source data 1/SUUR_MM-T_MM-I_333.jpg]

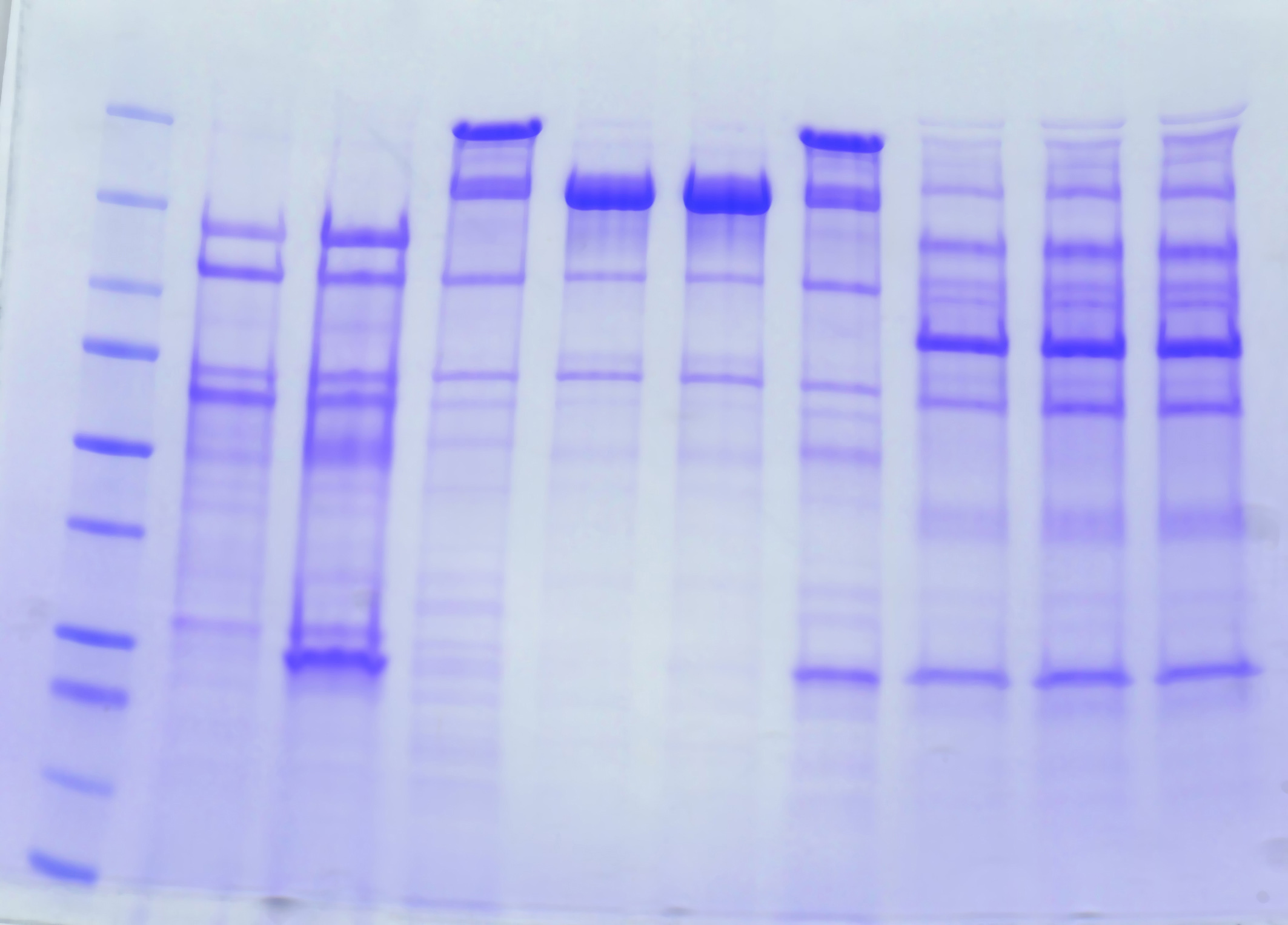

Supplement: Figure 3—figure supplement 1—source data 1. — Lane 1, protein size marker; lane 2, FLAG-SUUR, FLAG- purified; lane 3, FLAG-SUUR + EGG untagged, FLAG-purified; lane 4, WDE-FLAG + EGG untagged, FLAG-purified; lane 5, EGG-FLAG, FLAG-purified; lane 6, EGG-FLAG + His6-SUUR, FLAG-purified; lane 7, WDE-FLAG + EGG untagged + His6-SUUR, FLAG-purified; lane 8, His6-SUUR, Ni-NTA-purified; lane 9, EGG-FLAG + His6-SUUR, Ni-NTA-purified; lane 10, WDE-FLAG + EGG untagged + His6-SUUR, Ni-NTA-purified. All proteins were purified 72 hr post-infection. Prep amounts equivalent to ~20 ml Sf9 cultures were loaded in each lane. Cropped image encompassing lanes 1–4 (open box, dashed red line) was used for Figure 3—figure supplement 1A. [file elife-81828-fig3-figsupp1-data1.zip › Figure 3-figure supplement 1-source data 1/8_7_20.jpg]

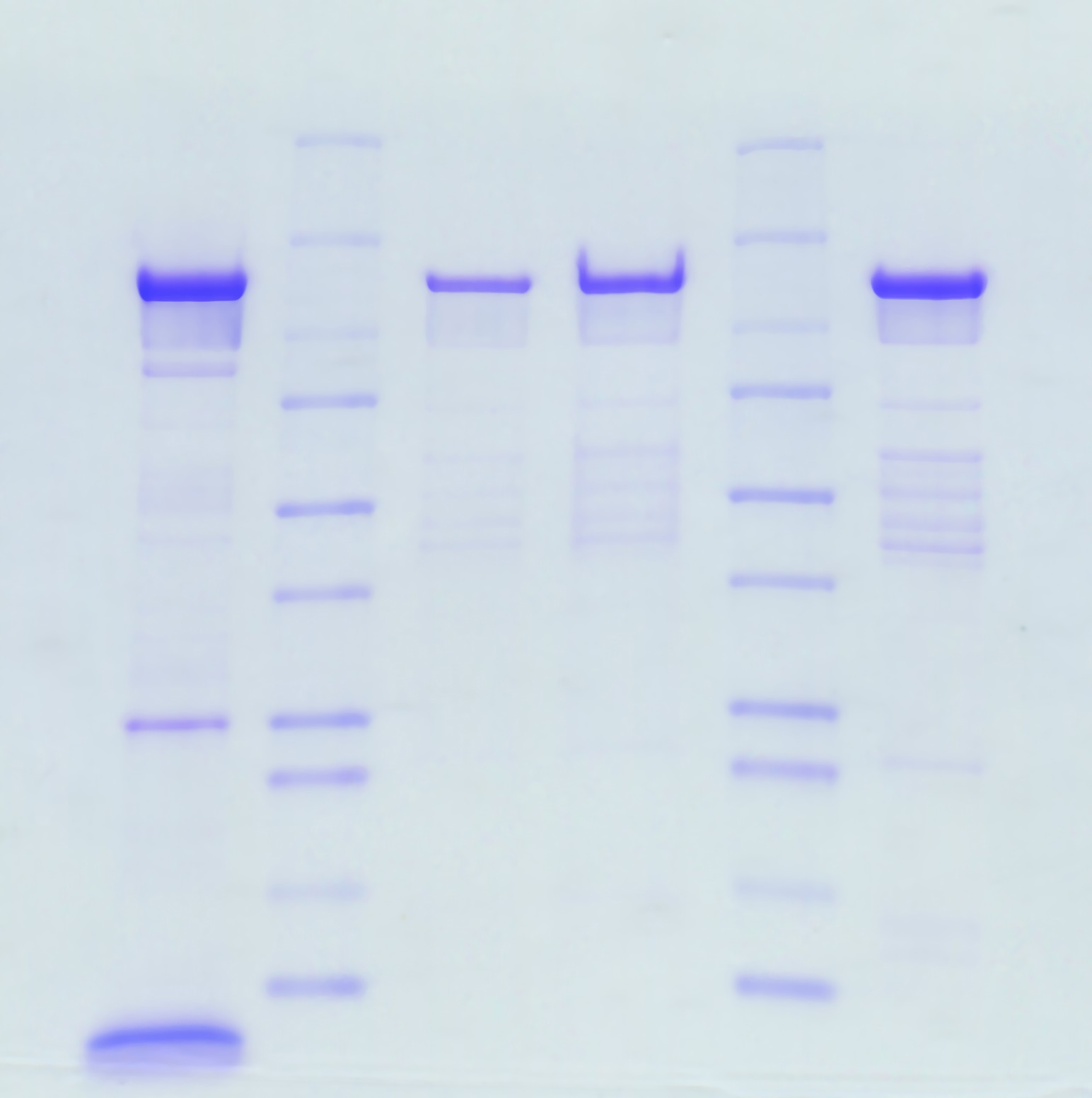

Supplement: Figure 3—figure supplement 1—source data 2. — Lanes 1, 7, and 10, protein size marker; lane 2, FLAG-SUUR(K59A), FLAG-purified; lane 3, FLAG-SUUR(K59R), FLAG-purified; lane 4, FLAG-SUUR wild-type, FLAG-purified; lane 5, His6-SUUR wild-type, Ni-NTA-purified. All proteins were purified 72 hr post-infection. Prep amounts equivalent to ~20 ml Sf9 cultures were loaded in each lane. Lane 6, FLAG-ISWI (Sf9 ells), FLAG-purified, 2 µg; lane 8, ISWI untagged (E. coli), chitin-purified, 0.5 µg; lane 9, ISWI untagged (E. coli), chitin-purified, 1 µg; lane 11, ISWI untagged (E. coli), chitin-purified, 2 µg. Cropped images encompassing lanes 1–2 and 10–11 (open boxes, dashed red line) were used for Figure 3—figure supplement 1B. [file elife-81828-fig3-figsupp1-data2.zip › Figure 3-figure supplement 1-source data 2/ISWIs_362.jpg]

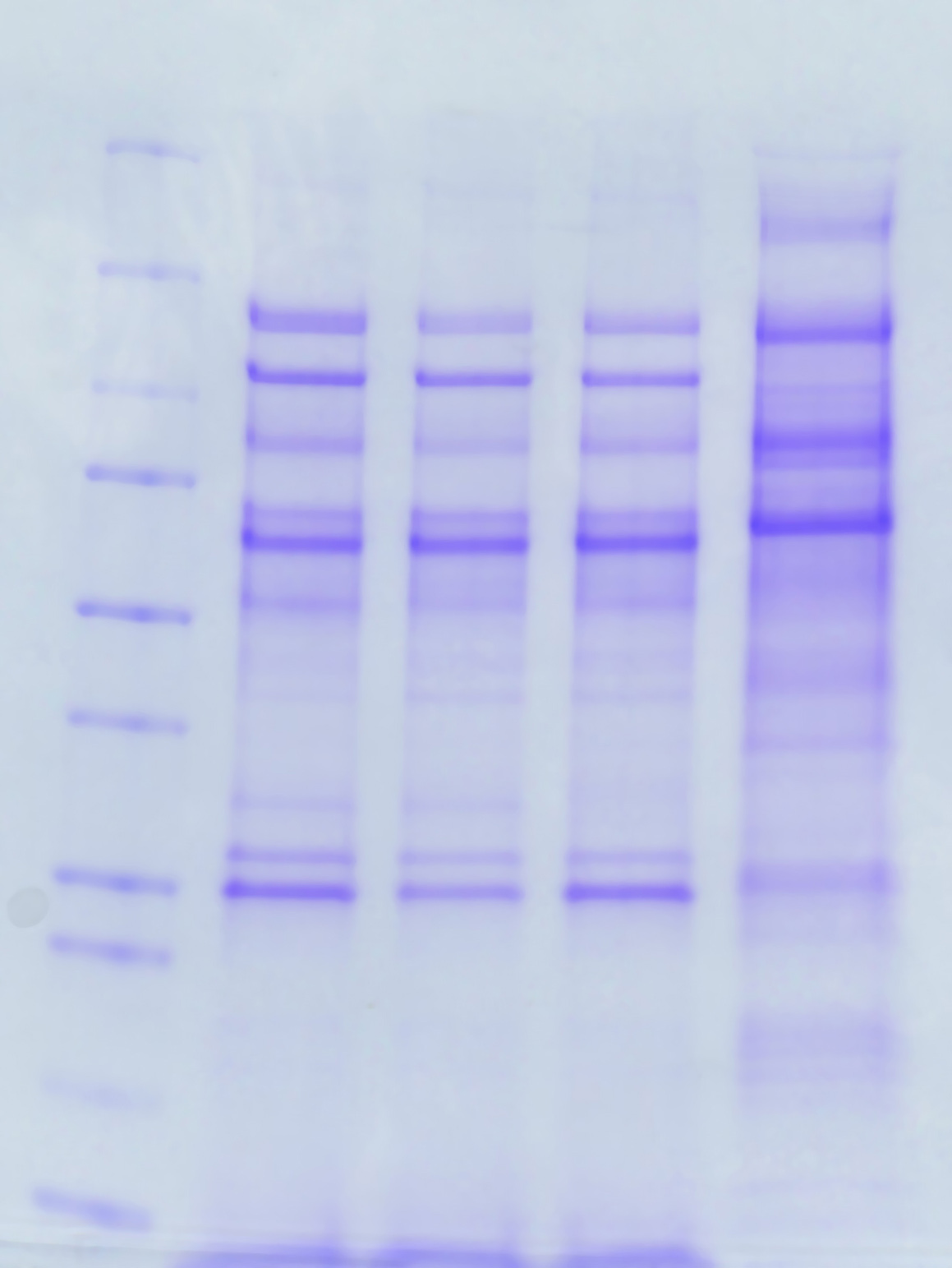

Supplement: Figure 3—figure supplement 1—source data 2. — Lanes 1, 7, and 10, protein size marker; lane 2, FLAG-SUUR(K59A), FLAG-purified; lane 3, FLAG-SUUR(K59R), FLAG-purified; lane 4, FLAG-SUUR wild-type, FLAG-purified; lane 5, His6-SUUR wild-type, Ni-NTA-purified. All proteins were purified 72 hr post-infection. Prep amounts equivalent to ~20 ml Sf9 cultures were loaded in each lane. Lane 6, FLAG-ISWI (Sf9 ells), FLAG-purified, 2 µg; lane 8, ISWI untagged (E. coli), chitin-purified, 0.5 µg; lane 9, ISWI untagged (E. coli), chitin-purified, 1 µg; lane 11, ISWI untagged (E. coli), chitin-purified, 2 µg. Cropped images encompassing lanes 1–2 and 10–11 (open boxes, dashed red line) were used for Figure 3—figure supplement 1B. [file elife-81828-fig3-figsupp1-data2.zip › Figure 3-figure supplement 1-source data 2/SUURs_6_24_21.jpg]

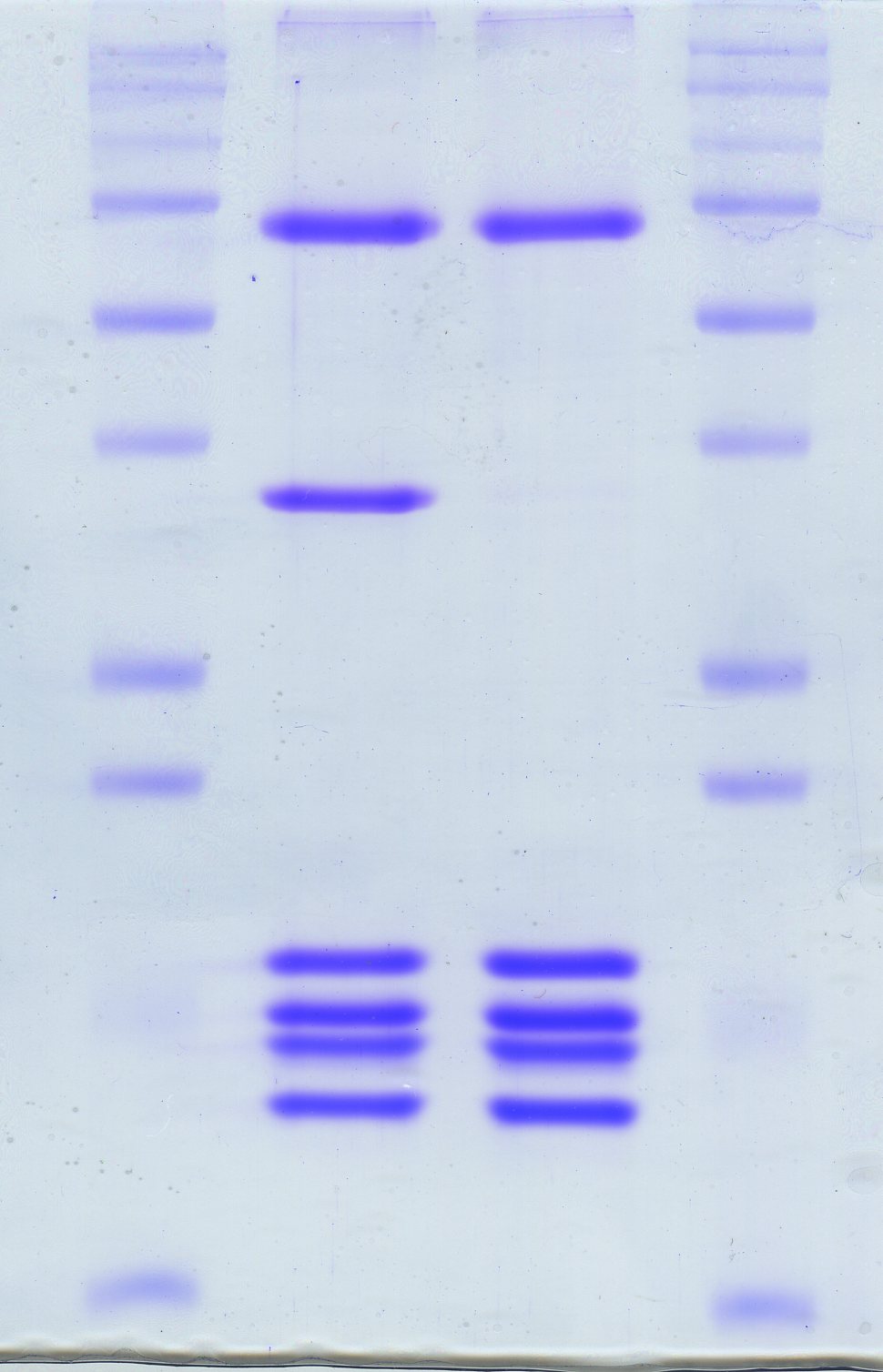

Supplement: Figure 3—figure supplement 1—source data 3. — Lanes 1 and 4, protein size marker; lane 2, oligonucleosomes, 2 µg DNA; lane 3, oligonucleosomes + H1, 2 µg DNA. Cropped image encompassing all lanes (open box, dashed red line) was used for Figure 3—figure supplement 1C. [file elife-81828-fig3-figsupp1-data3.zip › Figure 3-figure supplement 1-source data 3/Chr_H1_minus_plus_348.jpg]

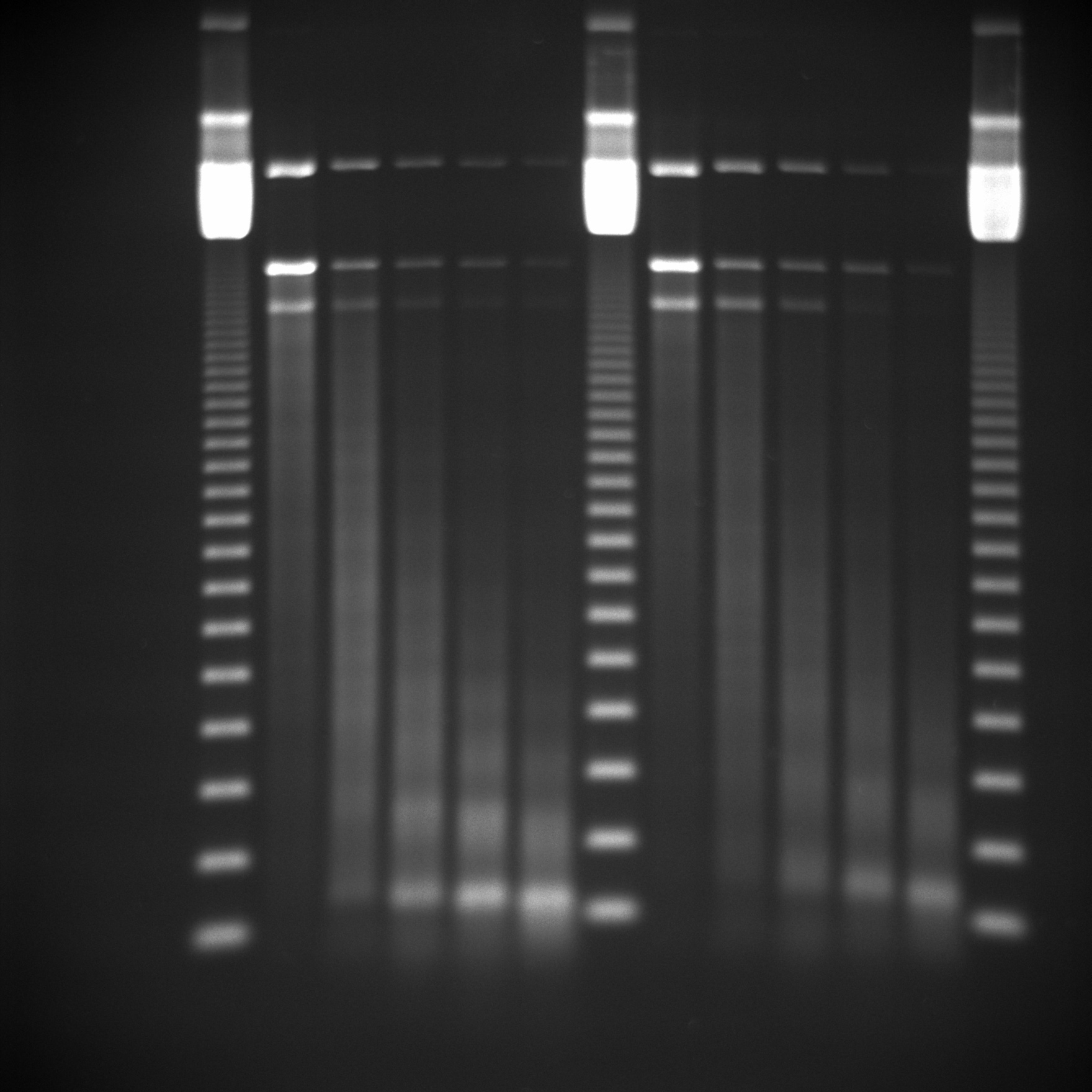

Supplement: Figure 3—figure supplement 1—source data 4. — Lanes 1, 7, and 13, 123-bp DNA ladder; lanes 2–6, oligonucleosomes, 250 ng DNA; lanes 8–12, oligonucleosomes + H1, 250 ng DNA; lanes 2 and 8, digested with 10-4 units MNase for 15 min at room temperature (RT); lanes 3 and 9, digested with 3•10-4 units MNase for 15 min at RT; lanes 4 and 10, digested with 10-3 units MNase for 15 min at RT; lanes 5 and 11, digested with 3•10-3 units MNase for 15 min at RT; lanes 6 and 12, digested with 10-2 units MNase for 15 min at RT. Cropped image encompassing all lanes (open box, dashed red line) was used for Figure 3—figure supplement 1D. [file elife-81828-fig3-figsupp1-data4.zip › Figure 3-figure supplement 1-source data 4/MNase_07-21-21.jpg]

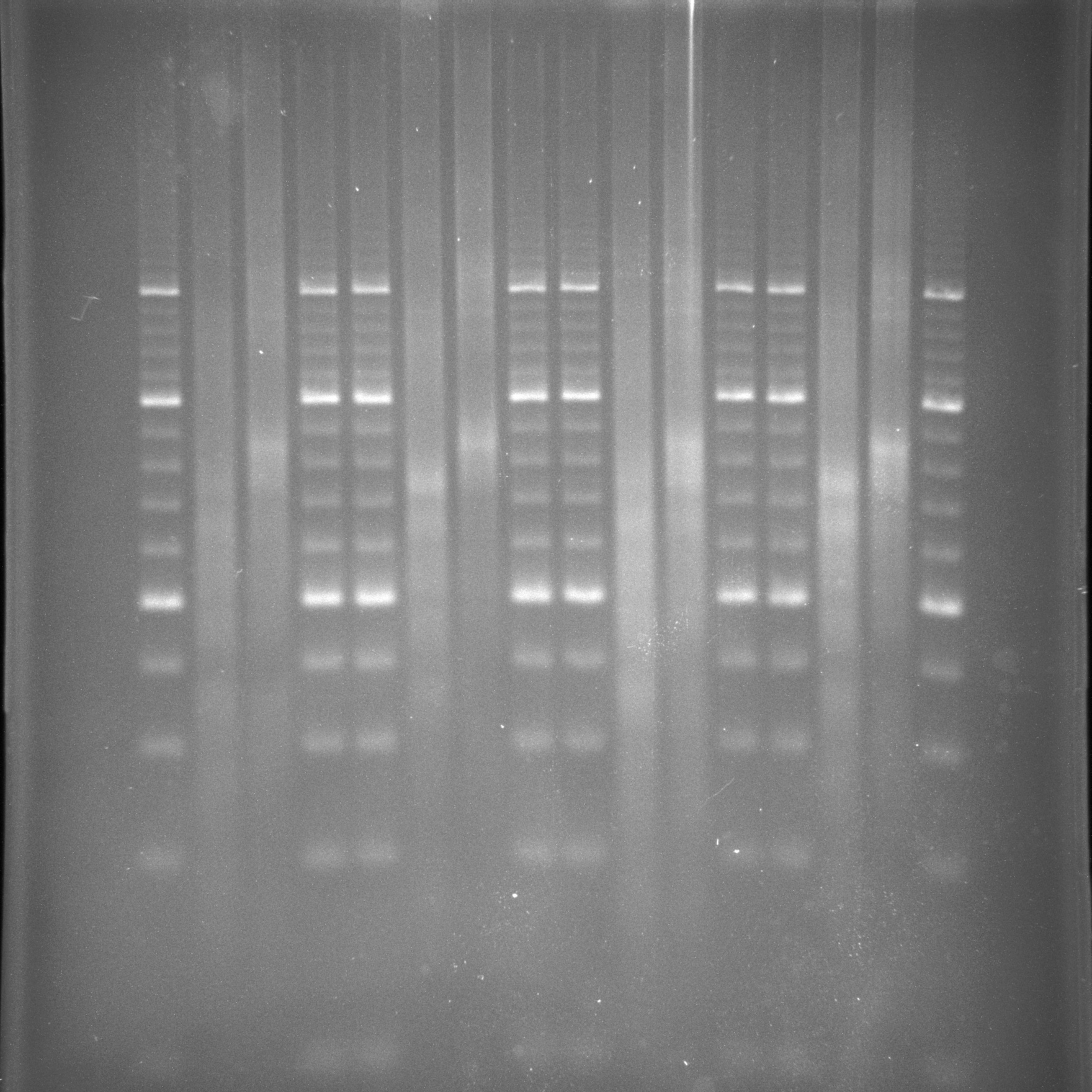

Supplement: Figure 3—figure supplement 1—source data 5. — Lanes 1, 4, 5, 8, 9, 12, 13, and 16, 20-bp DNA ladder; lanes 2, 6, 10, and 14, oligonucleosomes; lanes 3, 7, 11, and 15, oligonucleosomes +H 1; lanes 2 and 3, digested with 5•10-3 units MNase for 15 min at room temperature (RT); lanes 6 and 7, digested with 1.5•10-2 units MNase for 15 min at RT; lanes 10 and 11, digested with 10-2 units MNase for 15 min at RT; lanes 14 and 15, digested with 3•10-2 units MNase for 15 min at RT; lanes 2, 3, 6, and 7, 125 ng DNA; lanes 10, 11, 14, and 15, 250 ng DNA. Cropped image (open box, dashed red line) was used for Figure 3—figure supplement 1E. [file elife-81828-fig3-figsupp1-data5.zip › Figure 3-figure supplement 1-source data 5/ChrStop_07-30-21.jpg]

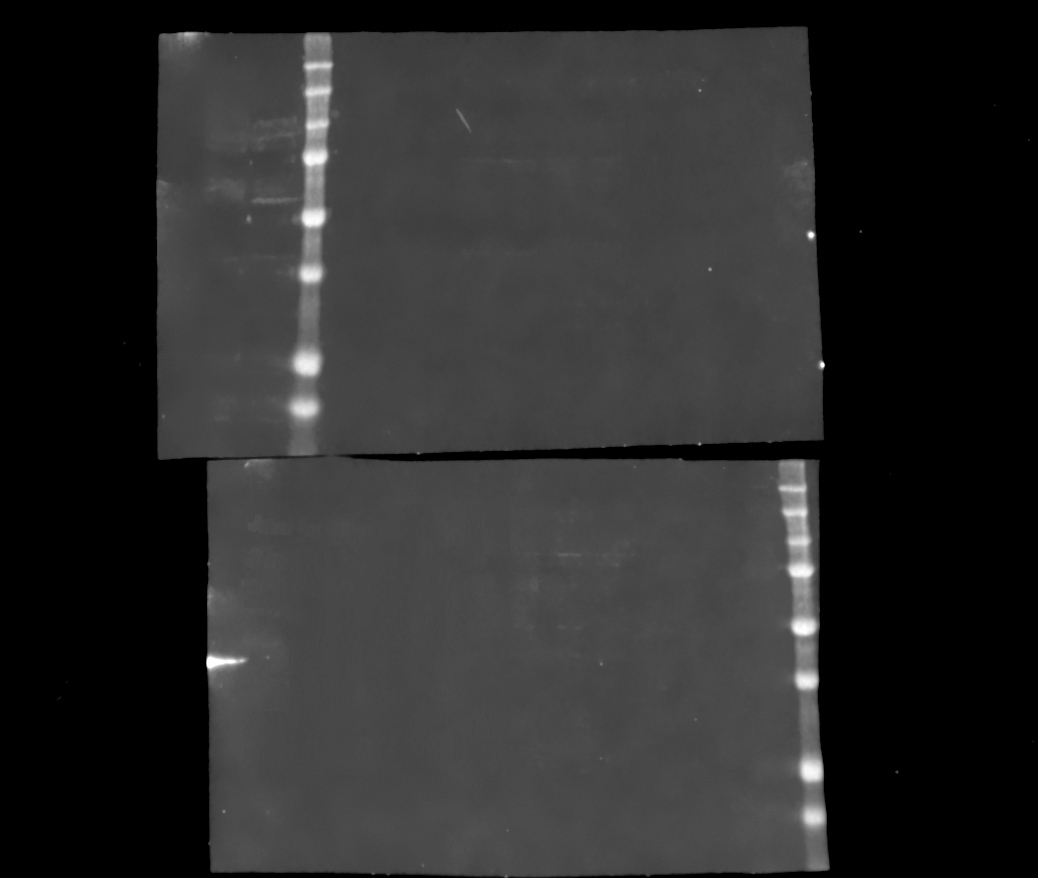

Supplement: Figure 4—figure supplement 2—source data 2. — Left panels, 700 nm channel (Odyssey Fc), rabbit anti-SUUR antibody and protein size marker; right panels, 800 nm channel (Odyssey Fc), guinea pig ModT antibody; top panels, Q Sepharose FF fractions: starting material, flow-through, marker, fractions 1–10; bottom panels, Q Sepharose FF fractions: fractions 11–22, marker. Cropped and spliced images encompassing all panels (open boxes, dashed red line) were used for Figure 4—figure supplement 2B. [file elife-81828-fig4-figsupp2-data2.zip › Figure 4-figure supplement 2-source data 2/0012719_01_700.jpg]

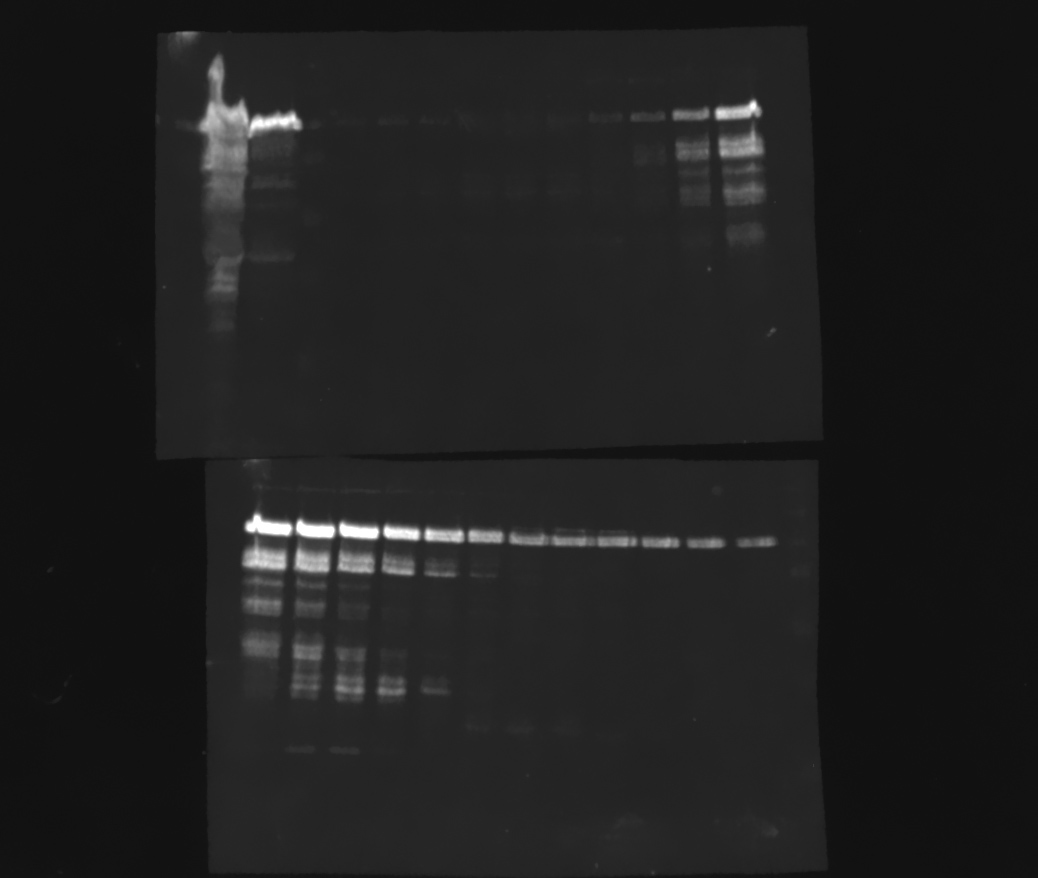

Supplement: Figure 4—figure supplement 2—source data 2. — Left panels, 700 nm channel (Odyssey Fc), rabbit anti-SUUR antibody and protein size marker; right panels, 800 nm channel (Odyssey Fc), guinea pig ModT antibody; top panels, Q Sepharose FF fractions: starting material, flow-through, marker, fractions 1–10; bottom panels, Q Sepharose FF fractions: fractions 11–22, marker. Cropped and spliced images encompassing all panels (open boxes, dashed red line) were used for Figure 4—figure supplement 2B. [file elife-81828-fig4-figsupp2-data2.zip › Figure 4-figure supplement 2-source data 2/0012719_01_800.jpg]

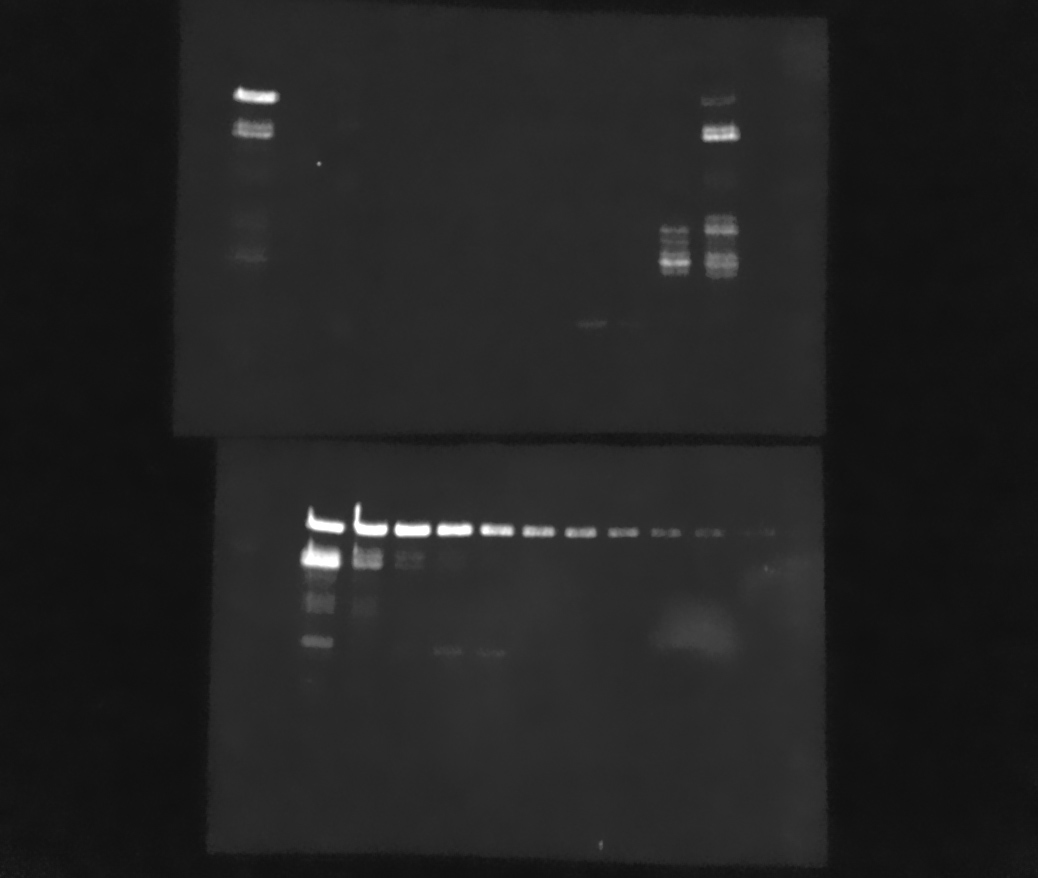

Supplement: Figure 4—figure supplement 2—source data 3. — Left panels, 700 nm channel (Odyssey Fc), rabbit anti-SUUR antibody and protein size marker; right panels, 800 nm channel (Odyssey Fc), guinea pig ModT antibody; top panels, Source 15S fractions: starting material, flow-through, marker, fractions 1–9, empty, marker; bottom panels, Source 15S fractions: marker, empty, fractions 10–20, marker. Cropped images encompassing all panels (open boxes, dashed red line) were used for Figure 4—figure supplement 2C. [file elife-81828-fig4-figsupp2-data3.zip › Figure 4-figure supplement 2-source data 3/0012727_01_800.jpg]

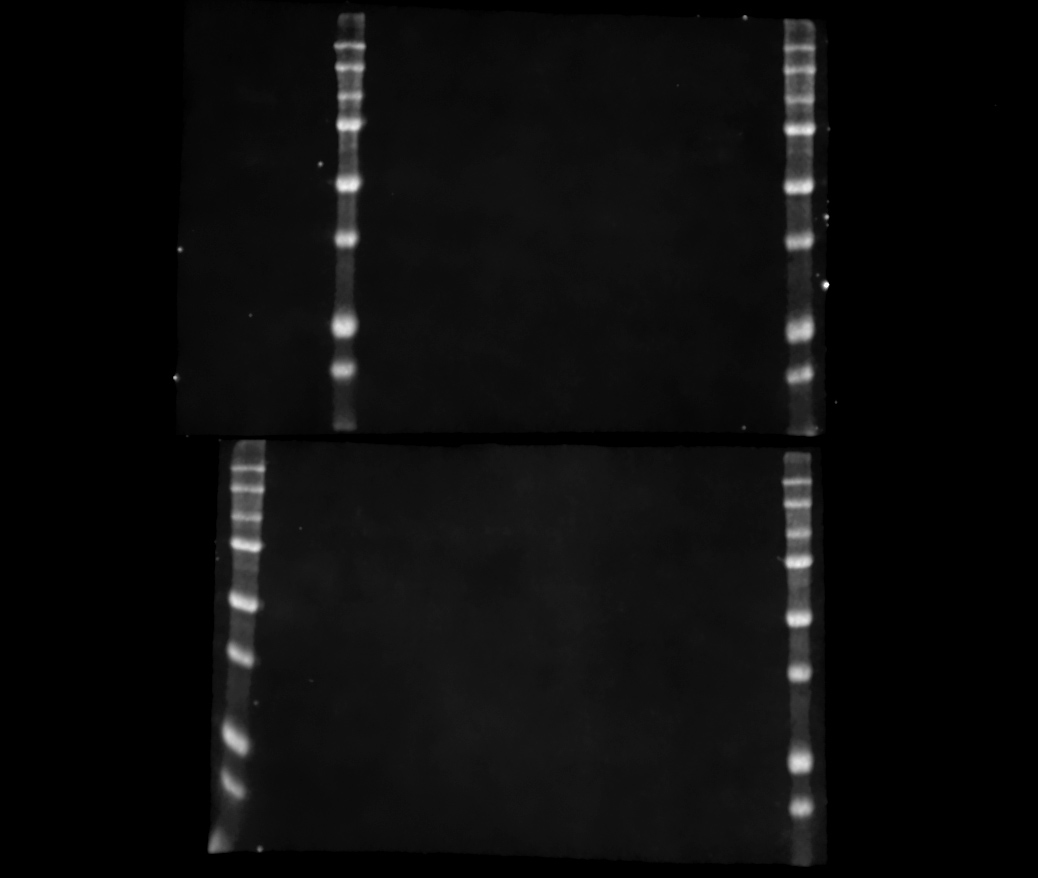

Supplement: Figure 4—figure supplement 2—source data 3. — Left panels, 700 nm channel (Odyssey Fc), rabbit anti-SUUR antibody and protein size marker; right panels, 800 nm channel (Odyssey Fc), guinea pig ModT antibody; top panels, Source 15S fractions: starting material, flow-through, marker, fractions 1–9, empty, marker; bottom panels, Source 15S fractions: marker, empty, fractions 10–20, marker. Cropped images encompassing all panels (open boxes, dashed red line) were used for Figure 4—figure supplement 2C. [file elife-81828-fig4-figsupp2-data3.zip › Figure 4-figure supplement 2-source data 3/0012727_01_700.jpg]

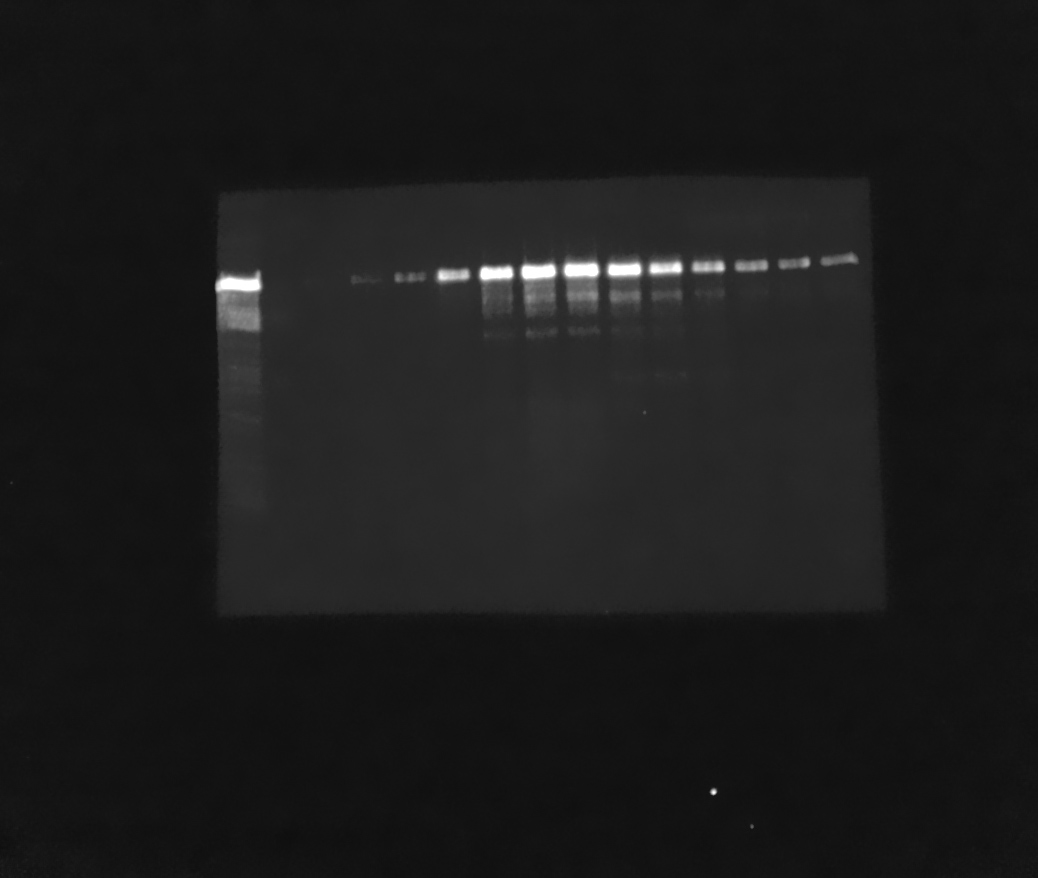

Supplement: Figure 4—figure supplement 2—source data 4. — Left panel, 700 nm channel (Odyssey Fc), protein size marker; right panels, 800 nm channel (Odyssey Fc), guinea pig ModT antibody. Superose 6 fractions: starting material, marker, fractions 1–13. Cropped images from both panels (open boxes, dashed red line) were used for Figure 4—figure supplement 2D. [file elife-81828-fig4-figsupp2-data4.zip › Figure 4-figure supplement 2-source data 4/0012753_01_800.jpg]

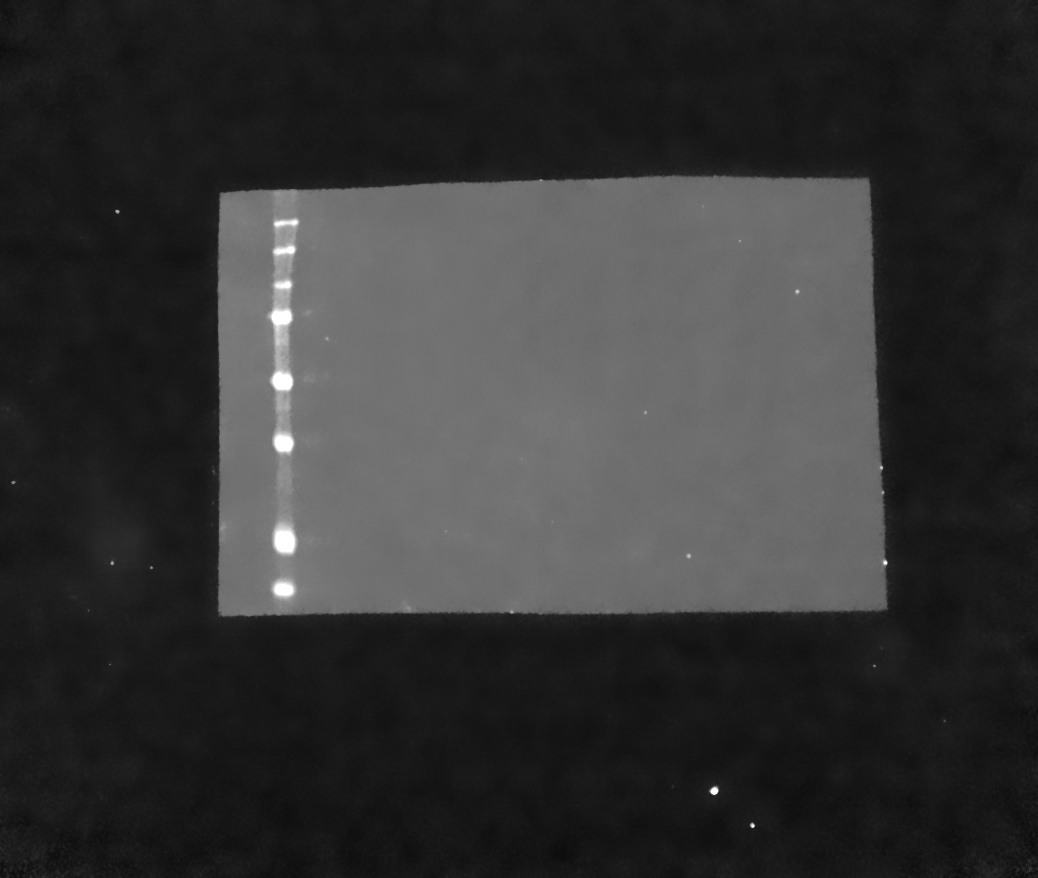

Supplement: Figure 4—figure supplement 2—source data 4. — Left panel, 700 nm channel (Odyssey Fc), protein size marker; right panels, 800 nm channel (Odyssey Fc), guinea pig ModT antibody. Superose 6 fractions: starting material, marker, fractions 1–13. Cropped images from both panels (open boxes, dashed red line) were used for Figure 4—figure supplement 2D. [file elife-81828-fig4-figsupp2-data4.zip › Figure 4-figure supplement 2-source data 4/0012753_01_700.jpg]

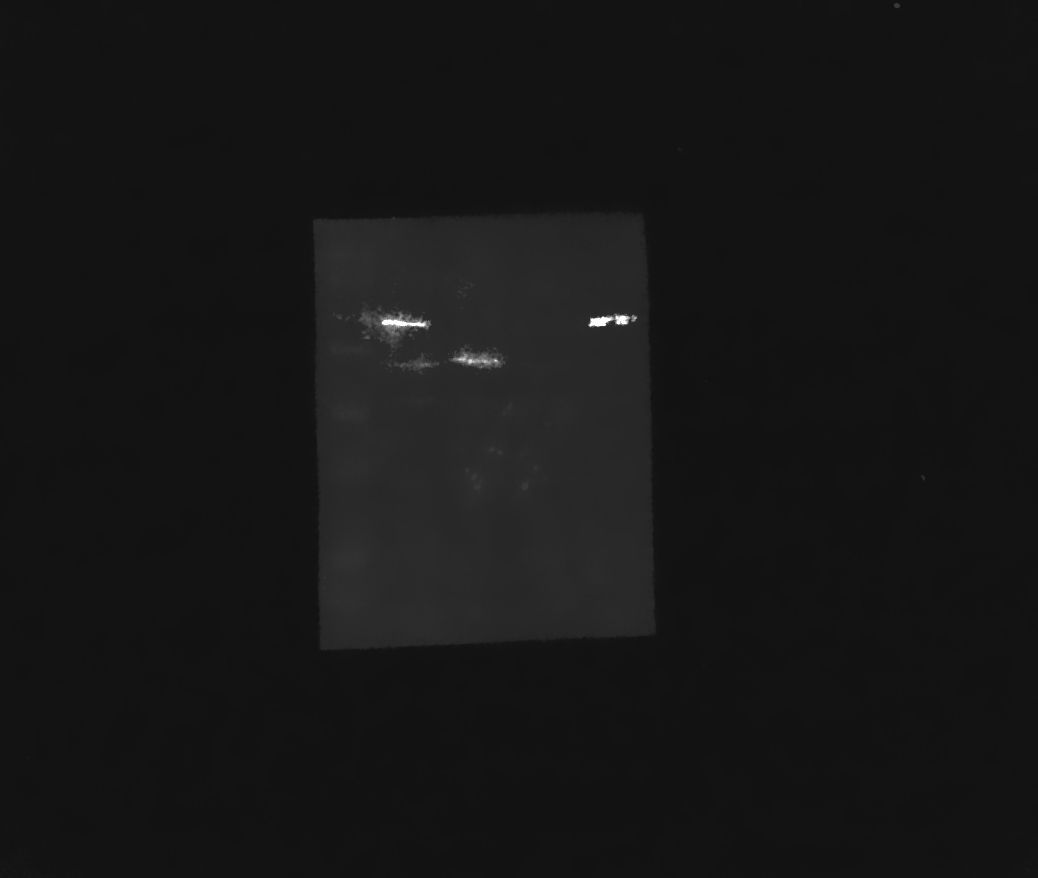

Supplement: Figure 4—figure supplement 3—source data 1. — Left panel, 700 nm channel (Odyssey Fc), mouse anti-tubulin antibody and protein size marker; right panels, 800 nm channel (Odyssey Fc), guinea pig ModT antibody. Lane 1, protein size marker; lane 2, L3 salivary glands, wild-type; lane 3, L3 salivary glands, mod(mdg4)u1; lane 4, L3 salivary glands, mod(mdg4)m9; lane 5, L3 salivary glands, SuURES. Cropped images from both panels (open boxes, dashed red line) overlayed in different colors (left panel, red; right panel, green) were used for Figure 4—figure supplement 3A. [file elife-81828-fig4-figsupp3-data1.zip › Figure 4-figure supplement 3-source data 1/0012691_01_800.jpg]

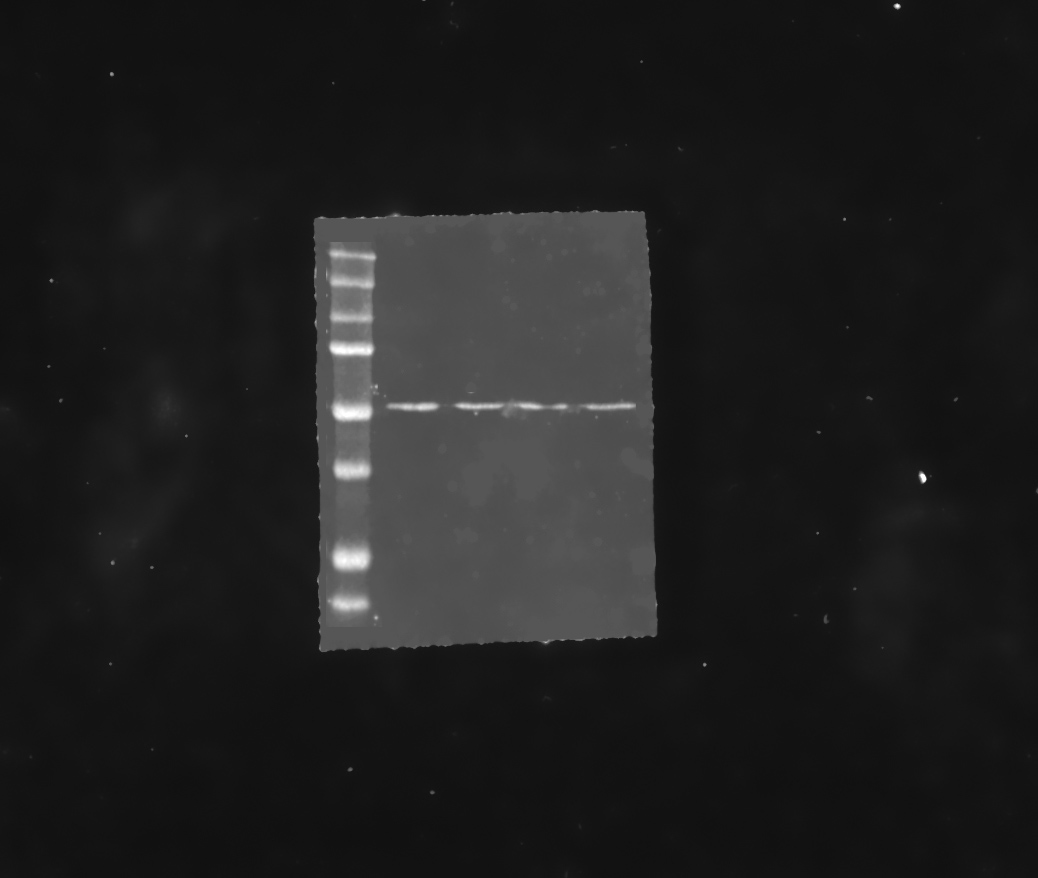

Supplement: Figure 4—figure supplement 3—source data 1. — Left panel, 700 nm channel (Odyssey Fc), mouse anti-tubulin antibody and protein size marker; right panels, 800 nm channel (Odyssey Fc), guinea pig ModT antibody. Lane 1, protein size marker; lane 2, L3 salivary glands, wild-type; lane 3, L3 salivary glands, mod(mdg4)u1; lane 4, L3 salivary glands, mod(mdg4)m9; lane 5, L3 salivary glands, SuURES. Cropped images from both panels (open boxes, dashed red line) overlayed in different colors (left panel, red; right panel, green) were used for Figure 4—figure supplement 3A. [file elife-81828-fig4-figsupp3-data1.zip › Figure 4-figure supplement 3-source data 1/0012691_01_700.jpg]
