# Supplementary material for: Drosophila SUMM4 complex couples insulator function and DNA replication control
Source: eLife. 2022 Dec 2;11:e81828. doi: 10.7554/eLife.81828 (PMC9917439; doi:10.7554/eLife.81828)
Supplement: Figure 4—figure supplement 2—source data 1. — The following FPLC column parameters were used for partial purification of an alternative complex of Mod(Mdg4)-67.2. HEG: 25 mM HEPES, pH 7.6, 0.1 mM EDTA, 10% glycerol, 0.02% NP-40, 1 mM DTT, 1 mM benzamidine, 0.4 mM PMSF; cv, column volume. [file elife-81828-fig4-figsupp2-data1.docx]

***Figure 4⎯figure supplement 2⎯source data 1.*** FPLC column parameters (***Figure 4⎯figure supplement 2A***). The following FPLC column parameters were used for partial purification of an alternative complex of Mod(Mdg4)-67.2. HEG: 25 mM HEPES, pH 7.6, 0.1 mM EDTA, 10% glycerol, 0.02% NP-40, 1 mM DTT, 1 mM benzamidine, 0.4 mM PMSF; *cv*, column volume.

| **Column** | **Q Sepharose FF** | **Source 15S** | **Superose 6** |
| --- | --- | --- | --- |
| Column volume, ml | 10 | 1 | 24 |
| Buffer A | HEG | HEG | HEG + 0.15 M NaCl |
| Buffer B | HEG + 1 M NaCl | HEG + 1 M NaCl | N/A |
| Starting material (SM) | nuclear extract | fxns 11-13 (Q) | fxn 11-13 (15S) |
| SM volume, ml | 20 | 8.5 | 0.6 |
| Diluted with | Buffer A | Buffer A | N/A |
| Dilution volume, ml | 10 | 25 | N/A |
| Equilibrate to, %B | 10% | 5% | 0% |
| Column wash, cv | 5 | 6 | N/A |
| Elution gradient | 10-100% | 5-100% | N/A |
| Elution volume, cv | 12 | 12 | 1.2 |
| Fraction volume, ml | 3 | 0.25 | 0.5 |
