# Supplementary material for: Drosophila SUMM4 complex couples insulator function and DNA replication control
Source: eLife. 2022 Dec 2;11:e81828. doi: 10.7554/eLife.81828 (PMC9917439; doi:10.7554/eLife.81828)
Supplement: Figure 7—source data 1. — Genomic coordinates indicate full amplicons, including the length of each primer. Coordinates refer to the BDGP R6/dm3 assembly. [file elife-81828-fig7-data1.docx]

***Figure 7⎯table supplement 1.*** Primer sequences used for qPCR. Genomic coordinates indicate full amplicons, including the length of each primer. Coordinates refer to the BDGP R6/dm3 assembly.

| **Cytological location 4C9-E3** | |  | **Cytological location 75B11-C2** | |
| --- | --- | --- | --- | --- |
| **Sequence** | **Genomic coordinates** |  | **Sequence** | **Genomic coordinates** |
| CCTCGATCGGTTTACATTCG  CCATAAACCCAAACGAGCTG | X:4,607,333..4,607,433 |  | ATTTGGACTGGGGCAGTTTC  CTGAAACACGGAAGTTGAGTCC | 3L:18,062,106..18,062,230 |
| CACATGGTGTCCTTGCATTC  GCCTAAACCAGCGATTCAAC | X:4,643,535..4,643,626 |  | AAAACACAAGCACATAGGCAAC  AGTTTCTGGCGTTGTATCCG | 3L:18,087,066..18,087,175 |
| GGGATGTGCTGCCTTTTATG  AGTTGCCACGACCAAAACTC | X:4,673,491..4,673,595 |  | GTGCACGGACGCGTATAATC  AAGTTAGCTCACGTGAGATGATG | 3L:18,164,427..18,164,499 |
| TGAAGGCCCTGGATGATAAG  TGGCATAGATATCGGTGTGC | X:4,706,888..4,706,995 |  | ACTATTATTTCTGGCTGGCTACG  GCCGGCTGCTACTTATGGC | 3L:18,188,845..18,188,948 |
| GGCTTGATTTTCGACTGCTC  AAAGGAAACAGCTCCGTGTG | X:4,742,052..4,742,153 |  | ATACAGATACAGCTCGCACTGG  AGTGGTGCCGATGGAAAAAC | 3L:18,214,103..18,214,210 |
| TTGCAGTGCCTCAAAGTCAG  ACCGACCAAAATCGAGACTG | X:4,774,644..4,774,740 |  | ACCACGCCCCTAAGCAAATAG  ATCTCGCCAGCTAAAGATCTCG | 3L:18,238,935..18,239,021 |
| CCTATCACCTGCCCATTTTG  TTACGTCCCTGGTTTCTTGC | X:4,826,332..4,826,430 |  | TGGGGCATTTTTGACGGTAG  GCTTTTAGCCTCGAGAAACCG | 3L:18,263,954..18,264,043 |
| AGCCATCCTGTTGCATCTTC  GCGCCAACAAATTCTCTCAG | X:4,856,456..4,856,547 |  | CTTGGCTCAGGTTTCCCTTC  AAAGGACGCCACAACAATGC | 3L:18,313,914..18,314,025 |
| ACCTCGCCAACATTACCAAC  AAACAACACGACGGCTCTTC | X:4,873,801..4,873,880 |  | ATCTCTCTGGGGCATCCAAG  CGCCAGCGCAGTTAAAAGTAAC | 3L:18,338,911..18,339,046 |
| AACTGCCCAAAGTGAAGGTG  GTTCAAGTGCAGCCAATGTG | X:4,893,272..4,893,370 |  | TGCACCAAGCTACACAATGG  CACAGGACTCCAAATTCTGCAC | 3L:18,364,090..18,364,232 |
| CGGCAAACACGACTACAATG  CAGTCGGATGCTGGTAGATATG | X:4,920,840..4,920,943 |  | AGTGATAGCGGAGTAACAGTGG  GTGGCGTGGATCCAACTTTATG | 3L:18,414,106..18,414,187 |
| AGCATGGACCCATCGATTAC  TTTCCCTGGGTAGCATTCAC | X:4,951,780..4,951,879 |  | TGCGCTAGTTCTCACCAACG  ACCAACTTAAGCACCAACTAAGG | 3L:18,439,417..18,439,489 |
| GAGATGCAAGATGCCACAAG  CCTTAGAGCGCTTCAATTCG | X:4,982,299..4,982,391 |  | ACGGGTGCCCTTAATGTTTAC  GGTCGTTGCCCATGTCTTTG | 3L:18,464,296..18,464,376 |
| AGGCAACCTGCAACTGAAAC  ACAATTGCGTACGTGAGCTG | X:5,009,757..5,009,859 |  | CAACCCTATCCATCCATCCATG  CAATCGGCCTAATTCACCCATG | 3L:18,491,978..18,492,057 |
| GTCTTGGAGTTGCCGTTTTG  TGCGCTGATCTCGTTAGATG | X:5,033,854..5,033,945 |  | ACATATTCGCCGACCAAGTG  ACACTAACACGTGCCCCTAAC | 3L:18,520,543..18,520,680 |
| CTAACCATCGCCAAATCCTC  CGTCCACAATTAGCTTGCAG | X:5,064,863..5,064,959 |  |  | |
|  |  |  | **Cytological location 86D9** | |
| TCCCTGCGACAACCTTTAAC  CTCCGTGACATGCTTGATTC | X:5,097,851..5,097,941 |  | **Sequence** | **Genomic coordinates** |
|  |  |  | TGGCGCCGCTTTCTTATTAG  AGAACAGGTTTGTGCGCTTG | 3R:11,261,333..11,261,450 |
|  |  |  |  |  |
